# Linkage Analysis and Map Construction in Genetic Populations of Clonal F_1_ and Double Cross

**DOI:** 10.1534/g3.114.016022

**Published:** 2015-01-15

**Authors:** Luyan Zhang, Huihui Li, Jiankang Wang

**Affiliations:** The National Key Facility for Crop Gene Resources and Genetic Improvement, Institute of Crop Science, and CIMMYT China Office, Chinese Academy of Agricultural Sciences, Beijing 100081, China

**Keywords:** clonal F_1_, double cross, recombination frequency, linkage analysis, map construction

## Abstract

In this study, we considered four categories of molecular markers based on the number of distinguishable alleles at the marker locus and the number of distinguishable genotypes in clonal F_1_ progenies. For two marker loci, there are nine scenarios that allow the estimation of female, male, and/or combined recombination frequencies. In a double cross population derived from four inbred lines, five categories of markers are classified and another five scenarios are present for recombination frequency estimation. Theoretical frequencies of identifiable genotypes were given for each scenario, from which the maximum likelihood estimates of one or more of the three recombination frequencies could be estimated. If there was no analytic solution, then Newton-Raphson method was used to acquire a numerical solution. We then proposed to use an algorithm in Traveling Salesman Problem to determine the marker order. Finally, we proposed a procedure to build the two haploids of the female parent and the two haploids of the male parent in clonal F_1_. Once the four haploids were built, clonal F_1_ hybrids could be exactly regarded as a double cross population. Efficiency of the proposed methods was demonstrated in simulated clonal F_1_ populations and one actual maize double cross. Extensive comparisons with software JoinMap4.1, OneMap, and R/qtl show that the methodology proposed in this article can build more accurate linkage maps in less time.

Plant species can be divided into three groups with respect to their sexual mating and asexual reproductive systems, *i.e.*, self-pollination, cross-pollination, and vegetative (or clonal or asexual) propagation (Allard 1999). An asexually propagated population consists of clones that are genetically identical to that of their parents. Reproduction by asexual propagation is common in higher plants, including nearly all fruit and nut trees such as strawberries, grapes, and pineapples; some field crops such as potatoes, sugarcane, yams, cassavas, and sweet potatoes; and many ornamental species (Allard 1999). Individual clonal plants usually show high heterozygosity. Once the superiority of any heterozygous clone is identified, this superiority can be protected and utilized by continued vegetative reproduction for a long period of time (Allard 1999).

Most clonal species have the problem of inbreeding depression, but hybridization between different clones, or even self-pollination of one clonal line, can produce seeds and therefore generate segregating clonal F_1_ progenies. Many genetic linkage studies have been conducted in clonal species, such as potatoes (Tanksley *et al.* 1992; van Os *et al.* 2006), cassavas (Fregene *et al.* 1997; Kunkeaw *et al.* 2010), sweet potatoes (Li *et al.* 2010), sugarcanes (Liu *et al.* 2010), populus (Zhang *et al.* 2000), pears (Yamamoto *et al.* 2002), apples (Hemmat *et al.* 1994), and pineapples (Carlier *et al.* 2004). Most studies focused on linkage map construction by adapting the clonal F_1_ progenies into inbred line–derived populations, such as pseudo-backcrosses or pseudo-testcrosses. This is a tedious procedure, and many less informative markers may not be used. For example, Hemmat *et al.* (1994) only considered three groups of markers in linkage map construction: those segregating as a result of heterozygosity in the female or male parent or in both parents. Many markers were discarded in estimation of recombination frequency before linkage map construction. Some studies on clonal species used the CP model (cross pollinators) in the software JoinMap (Stam 1993; van Ooijen 2006), which translates the clonal F_1_ progenies into a pseudo-backcross or pseudo-testcross population to estimate the recombination frequency in female and male parents.

Ritter *et al.* (1990) proposed a method of recombination frequency estimation between heterozygous parents based on RFLP markers, using part of the informative markers in the clonal F_1_ progenies. Ritter and Salamini (1996) considered more allelic configurations as an improvement of the previous work. Maliepaard *et al.* (1997) presented an overview of marker pair segregation configurations and then acquired the maximum likelihood estimators for the recombination frequency. Based on 18 cross types and the assumption that both parents had the same meiotic recombination, Wu *et al.* (2002a) proposed a methodology for linkage analysis in outcrossing species. Pairwise recombination frequency and linkage phase were estimated simultaneously by the posterior probabilities of the four different assignments conditional on the observed phenotype of the markers. Wu *et al.* (2002b) used the same algorithm in another study (Wu *et al.* 2002a), but considering the sex-specific recombination frequencies. Algorithms proposed by Wu *et al.* (2002a, b) were implemented in the R software (www.r-project.org) as a package called OneMap (Margarido *et al.* 2007). However, EM algorithm and Markov chains used in recombination frequency estimation and linkage phase determination were time-consuming. In addition, some configurations in the previous studies (Ritter and Salamini 1996; Maliepaard *et al.* 1997; Wu *et al.* 2002a, b) were identical in recombination frequency estimation. For example, Wu *et al.* (2002) gave 18 cross combinations based on the genotypes of the two parents. The first four each generates four genotypes, which can be properly identified in the progenies. They are identical when used in linkage analysis. Redundant configurations complicate the application of those methods in practical populations.

The R/qtl package could be used for linkage analysis in phase-known double cross (Broman *et al.* 2003), but it was not suitable for clonal F_1_ and phase-unknown double cross. It has been noted that software packages in R software were computationally slow and always failed to construct dense maps (van Ooijen 2011). Based on five segregation types of markers, van Ooijen (2011) proposed a Monte Carlo multipoint maximum likelihood algorithm to simultaneously estimate recombination frequency and determine marker order. An integrated map was generated by averaging lengths over anchored segments from two separate parental maps and by interpolating or extrapolating for markers segregating in only one parent. The methodology in van Ooijen (2011) was implemented in JoinMap4.1. The ordering algorithm used in JoinMap4.1 was called simulated annealing, which determines the best marker order by minimizing the sum of recombination frequencies in adjacent segments.

Genetic analysis methodology of clonal species is less investigated compared with self-pollinated and cross-pollinated species. In self-pollinated and cross-pollinated species, double crosses (or four-way crosses) can be made from four inbred lines to extend the genetic diversity in genetic studies and plant breeding. In clonal F_1_ and double cross, the number of alleles at each locus may be up to four. For each marker pair, there are four potential linkage phases in clonal F_1_. Once the linkage phase is determined, one clonal F_1_ can be viewed as a double cross population.

The unknown linkage phase and multiple alleles complicate recombination frequency estimation in clonal F_1_ and double cross populations. Our objectives in this study were: (1) to identify and classify informative markers based on the number of distinguishable alleles and the number of distinguishable genotypes; (2) to derive the theoretical frequencies of identifiable genotypes for each scenario of marker pairs and maximum likelihood estimates of recombination frequencies; (3) to build the female, male, and combined linkage maps; (4) to build the four haploids of the female and male parents based on the estimated recombination frequencies and the combined linkage map; and (5) to demonstrate the advantage of the proposed methods in comparison with other software.

## Materials and Methods

### Marker categories and coding criteria in clonal F_1_ progenies

**Figure 1:**
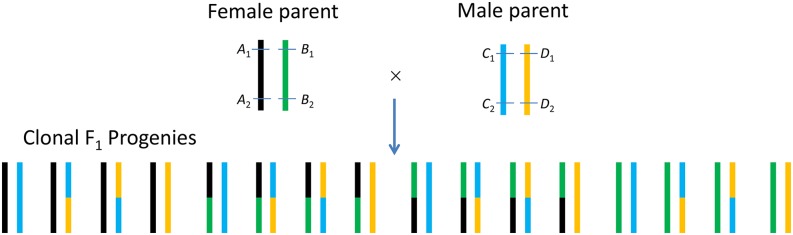
Diagram of the development of a clonal F_1_ population derived from two clonal parents, which are highly heterozygous at a large number of loci, assuming locus 1 and locus 2 were two linked polymorphism markers. *A*_1_, *B*_1_, *C*_1_, and *D*_1_ were the four alleles at marker locus 1. *A*_2_, *B*_2_, *C*_2_, and *D*_2_ were the four alleles at marker locus 2. It should be noted that the female parent could have genotype *A*_1_*B*_1_/*A*_2_*B*_2_ or *A*_1_*B*_2_/*A*_2_*B*_1_, and the male parent could have genotype *C*_1_*D*_1_/*C*_2_*D*_2_ or *C*_1_*D*_2_/*C*_2_*D*_1_.

**Figure 2:**
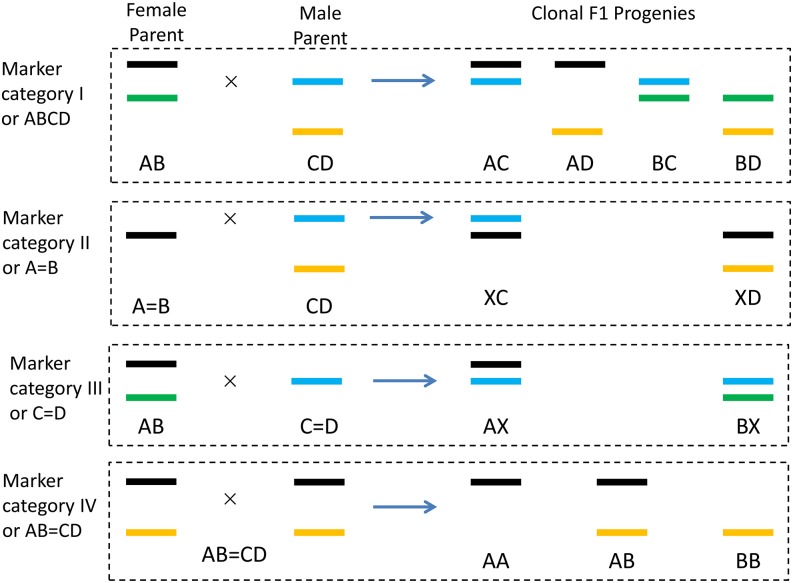
Four categories of polymorphism markers that can be used in genetic study in clonal F_1_ populations. In category I or ABCD, each marker shows four identifiable alleles between the two clonal parents, represented by *A*, *B*, *C*, and *D* (see the four different colors in Figure 1). In the clonal population, four genotypes can be identified, represented by *AC*, *AD*, *BC*, and *BD*. In category II or A = B, one allele can be seen in the female parent and two alleles can be seen in male parent. In the clonal population, only two genotypes can be identified, represented by *XC* and *XD*, where *X* can be either *A* or *B*. In category III or C = D, two alleles can be seen in the female parent and one allele can be seen in male parent. The two identifiable genotypes in the clonal population are represented by *AX* and *BX*, where *X* can be either *C* or *D*. In category IV or AB = CD, both clonal parents show the same two heterozygous genotype. The two alleles in parents are represented by *A* and *B*, and three genotypes in their progenies are represented by *AA*, *AB*, and *BB*.

Genetic studies in clonal species are normally conducted in F_1_ hybrids of two clonal parents, one used as female and the other used as male (Figure 1). The two parents are normally heterozygous and unrelated or less related in genetics, and therefore may have up to four identifiable alleles at each polymorphism locus. In this study, *A* and *B* were used to represent the two potential alleles in the female parent; *C* and *D* represented the two potential alleles in the male parent, as indicated at two loci in Figure 1. Based on the actual number of identifiable alleles in the two parents and the actual number of identifiable genotypes in the F_1_ progenies, each marker locus can be classified into four categories (Figure 2).

Category I (or ABCD) represents the case of fully informative markers. By fully informative, we mean the four genotypes at one locus in progenies can be clearly identified. In other words, the two alleles in any clonal progeny can be traced back to its female and male parents (Figure 2). For category I markers, two alleles can be identified in either parent. The four genotypes in progenies are coded as *AC*, *AD*, *BC*, and *BD* (Figure 2). When no distortion occurs, the four genotypes will follow the Mendelian ratio of 1:1:1:1. However, it is possible that one female allele is the same as one male allele. For example, when allele *A* is equal to allele *C* at a marker locus, there is no problem assigning the two alleles in a progeny to the two parents. This marker is still classified as category I.

Category II (or A = B) represents the case of male polymorphism markers. By male polymorphism markers, we mean they show no polymorphism in the female parent, but they show polymorphism in the male parent. For category II markers, only two genotypes can be observed in the clonal F_1_ progenies (Figure 2). Genotypes *AC* and *BC* cannot be separated; neither can genotypes *AD* and *BD*. In this category, *XC* is used to code genotypes *AC* and *BC*; *XD* is used to code genotypes *AD* and *BD*, where *X* stands for either allele *A* or allele *B* (Figure 2). When no distortion occurs, the two genotypes will follow the Mendelian ratio of 1:1.

Category III (or C = D) represents the case of female polymorphism markers. By female polymorphism markers, we mean they show polymorphism in the female parent, but they show no polymorphism in the male parent. For category III markers, only two genotypes can be observed in the clonal F_1_ progenies (Figure 2). Genotypes *AC* and *AD* cannot be separated; neither can genotypes *BC* and *BD*. In this category, *AX* is used to code genotypes *AC* and *AD*; *BX* is used to code genotypes *BC* and *BD*, where *X* stands for either allele *C* or *D* (Figure 2). When no distortion occurs, the two genotypes will follow the Mendelian ratio of 1:1.

Category IV (or AB = CD) represents the case of co-dominant markers. By co-dominant markers, we mean they show the same polymorphism pattern in both female and male parents, similar to an F_2_ population derived from two inbred parents in self-pollinated and cross-pollinated species. For category IV markers, three genotypes can be observed in the clonal F_1_ progenies, which are coded by *AA*, *AB*, and *BB*, respectively (Figure 2). When no distortion occurs, the three genotypes will follow the Mendelian ratio of 1:2:1.

Missing marker types are common in most genetic populations (Zhang *et al.* 2010). In these four categories, any missing values of marker type are coded as *XX*.

### Nine scenarios between two loci in recombination frequency estimation in clonal F_1_ progenies

Assuming that locus 1 and locus 2 are two linked polymorphism markers, falling into one of the four categories in Figure 2, let *A*_1_, *B*_1_, *C*_1_, and *D*_1_ be the four alleles at locus 1 and let *A*_2_, *B*_2_, *C*_2_, and *D*_2_ be the four alleles at locus 2. Recombination frequencies in the female and male parents were denoted as *r_F_* and *r_M_*, which can be used to construct the female and male linkage maps, respectively. The combined recombination frequency is denoted as *r*, which can be used to construct the combined map. Due to the symmetry of marker pairs, we consider nine scenarios between two loci in clonal F_1_ populations where at least one of the above three recombination frequencies can be estimated (Table 1). Scenario 1 represents the most ideal situation where all recombination frequencies can be properly estimated. If one locus is category II and the other one is category III (not included in Table 1), then the four genotypes at the two loci have an equal theoretical frequency of 0.25. In this scenario, none of *r_F_*, *r_M_*, and *r* can be estimated.

**Table 1:** The nine scenarios between two linked loci in the clonal F_1_ population for estimating the recombination frequency

Scenario	Marker Category		Recombination Frequency		
	Locus 1	Locus 2	*r_F_*	*r_M_*	*r*
1	I (ABCD)	I (ABCD)	√	√	√
2	I (ABCD)	II (A = B)		√	
3	I (ABCD)	III (C = D)	√		
4	I (ABCD)	IV (AB = CD)	1/2√	1/2√	√
5	II (A = B)	II (A = B)		√	
6	II (A = B)	IV(AB = CD)		√	
7	III (C = D)	III (C = D)	√		
8	III (C = D)	IV (AB = CD)	√		
9	IV (AB = CD)	IV (AB = CD)			√

The symbol √ is used to indicate that recombination frequency *r_F_*, *r_M_*, or *r* could be estimated, and 1/2 is used to indicate that only half of the observed samples are used in estimating recombination frequency. When one marker is category II and the other one marker is category III, recombination frequency between them cannot be estimated and therefore it is not included.

When one locus is category II, there is no polymorphism in the female parent; therefore, *r_F_* cannot be estimated (Table 1). Similarly, when one locus is category III, there is no polymorphism in the male parent; therefore, *r_M_* cannot be estimated (Table 1). In scenario 4, only half of samples can be used to estimate *r_F_* and *r_M_* (Table 1). In scenario 9, the linkage information in the two parents is confounded. It is impossible to estimate *r_F_* and *r_M_*. However, the combined *r* can still be estimated (Table 1).

### Linkage phases between two loci to be determined in clonal F_1_ progenies

In clonal F_1_ progenies, linkage phases of the two loci in both parents are unknown before linkage analysis. When marker loci 1 and 2 show polymorphism in the female parent, *A*_1_ and *B*_1_ are randomly assigned for the two alleles at locus 1, and *A*_2_ and *B*_2_ are randomly assigned for the two alleles at locus 2. Genotype of the female parent can be either *A*_1_*A*_2_/*B*_1_*B*_2_ or *A*_1_*B*_2_/*B*_1_*A*_2_, where “/” was used to separate the two homologous chromosomes. For both phases, genotype of the female parent is *A*_1_*B*_1_ at locus 1 and is *A*_2_*B*_2_ at locus 2. The same situation applies in the male parent. Genotype of the male parent can be either *C*_1_*C*_2_/*D*_1_*D*_2_ or *C*_1_*D*_2_/*D*_1_*C*_2_. For both phases, genotype of the female parent is *C*_1_*D*_1_ at locus 1 and *C*_2_*D*_2_ at locus 2. Linkage phases in both parents are to be determined by linkage analysis.

Taking the female parent as an example, the four female gametes are *A*_1_*A*_2_, *A*_1_*B*_2_, *B*_1_*A*_2_, and *B*_1_*B*_2_, and their frequencies are represented by $\frac{1}{2}(1-r_{F})$, $\frac{1}{2}r_{F}$, $\frac{1}{2}r_{F}$, and $\frac{1}{2}(1-r_{F})$ (see Supporting Information, Table S1). In the case of genotype *A*_1_*A*_2_/*B*_1_*B*_2_, gametes *A*_1_*A*_2_ and *B*_1_*B*_2_ are the two noncrossover (or parental) types with a frequency of $\left(1-r_{F}\right)$, and *A*_1_*B*_2_ and *B*_1_*A*_2_ are the two crossover (or recombination, or nonparental) types with a frequency of $r_{F}$. The estimated *r_F_* will be lower than 0.5 if the two loci are linked. In the case of genotype *A*_1_*B*_2_/*B*_1_*A*_2_, gametes *A*_1_*A*_2_ and *B*_1_*B*_2_ are the two crossover types with a frequency of $\left(1-r_{F}\right)$, and *A*_1_*B*_2_ and *B*_1_*A*_2_ are the two noncrossover types with a frequency of $r_{F}$. The estimated *r_F_* will be more than 0.5 when the two loci are linked. Obviously, whether the estimated *r_F_* is less or more than 0.5 could help to determine the linkage phase and genotype of the female parent. Similarly, whether the estimated *r_M_* is less or more than 0.5 could help to determine the linkage phase and genotype of the male parent.

Therefore, linkage phases and genotypes of both parents can be determined by their estimated recombination frequencies, respectively. If estimated *r_F_* is less than 0.5, then the female parent will be in linkage phase *A*_1_*A*_2_/*B*_1_*B*_2_; otherwise, it will be in linkage phase *A*_1_*B*_2_/*B*_1_*A*_2_. If estimated *r_M_* is less than 0.5, then the male parent will be in linkage phase *C*_1_*C*_2_/*D*_1_*D*_2_; otherwise, it will be in linkage phase *C*_1_*D*_2_/*D*_1_*C*_2_.

Considering the two phases to be determined in both parents together, four potential linkage phases of the two parents can be defined. In phase I, the female parent has genotype *A*_1_*A*_2_/*B*_1_*B*_2_ and the male parent has genotype *C*_1_*C*_2_/*D*_1_*D*_2_. In phase II, the female parent has genotype *A*_1_*A*_2_/*B*_1_*B*_2_ and the male parent has genotype *C*_1_*D*_2_/*D*_1_*C*_2_. In phase III, the female parent has genotype *A*_1_*B*_2_/*B*_1_*A*_2_ and the male parent has genotype *C*_1_*C*_2_/*D*_1_*D*_2_. In phase IV, the female parent has genotype *A*_1_*B*_2_/*B*_1_*A*_2_ and the male parent has genotype *C*_1_*D*_2_/*D*_1_*C*_2_. The four phases will be used later for some scenarios in estimating the combined recombination frequency *r*, to make sure the estimated *r* is less than 0.5, and the estimation will not be affected by the linkage information confounding in one or both parents.

### Recombination frequency estimation in scenario 1 in clonal F_1_ progenies

We begin with the most ideal situation where locus 1 has four identifiable genotypes *A*_1_*C*_1_, *A*_1_*D*_1_, *B*_1_*C*_1_, and *B*_1_*D*_1_, and locus 2 has four identifiable genotypes *A*_2_*C*_2_, *A*_2_*D*_2_, *B*_2_*C*_2_, and *B*_2_*D*_2_. The first row and first column of Table S1 show the four female and male gametes and their frequencies, from which we can easily derive theoretical frequencies of the 16 identifiable genotypes at the two linked loci. For convenience, the 16 genotypes were rearranged in Table 2, and sample sizes of the 16 genotypes were represented by *n*_1_, *n*_2_, …, and *n*_16_. Based on theoretical frequencies and sample sizes in Table 2, the likelihood function (*L*) and logarithm likelihood (log*L*) can be constructed in Equation (1).

**Table 2:** Theoretical frequencies of the 16 identifiable genotypes in the clonal F_1_ population at two linked loci

Genotype	Locus 1	Locus 2	Frequency	Sample Size
1	*A*_1_*C*_1_	*A*_2_*C*_2_	$\frac{1}{4}(1-r_{F})(1-r_{M})$	*n*_1_
2	*A*_1_*C*_1_	*A*_2_*D*_2_	$\frac{1}{4}(1-r_{F})r_{M}$	*n*_2_
3	*A*_1_*D*_1_	*A*_2_*C*_2_	$\frac{1}{4}(1-r_{F})r_{M}$	*n*_3_
4	*A*_1_*D*_1_	*A*_2_*D*_2_	$\frac{1}{4}(1-r_{F})(1-r_{M})$	*n*_4_
5	*A*_1_*C*_1_	*B*_2_*C*_2_	$\frac{1}{4}r_{F}(1-r_{M})$	*n*_5_
6	*A*_1_*C*_1_	*B*_2_*D*_2_	$\frac{1}{4}r_{F}r_{M}$	*n*_6_
7	*A*_1_*D*_1_	*B*_2_*C*_2_	$\frac{1}{4}r_{F}r_{M}$	*n*_7_
8	*A*_1_*D*_1_	*B*_2_*D*_2_	$\frac{1}{4}r_{F}(1-r_{M})$	*n*_8_
9	*B*_1_*C*_1_	*A*_2_*C*_2_	$\frac{1}{4}r_{F}(1-r_{M})$	*n*_9_
10	*B*_1_*C*_1_	*A*_2_*D*_2_	$\frac{1}{4}r_{F}r_{M}$	*n*_10_
11	*B*_1_*D*_1_	*A*_2_*C*_2_	$\frac{1}{4}r_{F}r_{M}$	*n*_11_
12	*B*_1_*D*_1_	*A*_2_*D*_2_	$\frac{1}{4}r_{F}(1-r_{M})$	*n*_12_
13	*B*_1_*C*_1_	*B*_2_*C*_2_	$\frac{1}{4}(1-r_{F})(1-r_{M})$	*n*_13_
14	*B*_1_*C*_1_	*B*_2_*D*_2_	$\frac{1}{4}(1-r_{F})r_{M}$	*n*_14_
15	*B*_1_*D*_1_	*B*_2_*C*_2_	$\frac{1}{4}(1-r_{F})r_{M}$	*n*_15_
16	*B*_1_*D*_1_	*B*_2_*D*_2_	$\frac{1}{4}(1-r_{F})(1-r_{M})$	*n*_16_

Four alleles can be clearly identified at each of the two linked loci (scenario 1 in Table 1). *A*_1_, *B*_1_, *C*_1_, and *D*_1_ are the four alleles at locus 1. *A*_2_, *B*_2_, *C*_2_, and *D*_2_ are the four alleles at locus 2. Recombination frequencies in the female and male parents are denoted as *r_F_* and *r_M_*, respectively. The last column gives the symbol of observed sample size of each genotype

$$\begin{array}{c} L=\frac{n!}{n_{1}!\cdots n_{16}!}{\left[\frac{1}{4}\left(1-r_{F}\right)\left(1-r_{M}\right)\right]}^{n_{1}+n_{4}+n_{13}+n_{16}}{\times [\frac{1}{4}\left(1-r_{F}\right)r_{M}]}^{n_{2}+n_{3}+n_{14}+n_{15}}{\left[\frac{1}{4}r_{F}\left(1-r_{M}\right)\right]}^{n_{5}+n_{8}+n_{9}+n_{12}}{\times [\frac{1}{4}r_{F}r_{M}]}^{n_{6}+n_{7}+n_{10}+n_{11}}\\ \log\;L=C+\left(n_{1:4}+n_{13:16}\right)\log\left(1-r_{F}\right)+n_{5:12}\log\;r_{F}+\left(n_{1}+n_{4:5}+n_{8:9}+n_{12:13}+n_{16}\right)\times \log\left(1-r_{M}\right)+\left(n_{2:3}+n_{6:7}+n_{10:11}+n_{14:15}\right)\log\;r_{M}, \end{array}$$ (1)

where *C* is a constant independent of the unknown recombination frequencies. The maximum likelihood estimates (MLE) of recombination frequencies can be calculated either by solving the likelihood equation (*i.e.*, $\frac{d\log\;L}{dr}=0$) or by some approximate algorithms when there is no analytic solution to the likelihood equation. From Equation (1), MLE of *r_F_* and *r_M_* can be directly calculated from Equation (2).

$${\hat{r}}_{F}=\frac{n_{5:12}}{n},{\hat{r}}_{M}=\frac{n_{2:3}+n_{6:7}+n_{10:11}+n_{14:15}}{n},$$ (2)

where *n_i_* is the observed sample size for the *i*th genotype (Table 2), *n_i_*_:_*_j_* is the sum of *n_i_* to *n_j_*, and *n* is the total sample size (*i.e.*, *n*=*n*_1:16_).

Define the estimate of the combined recombination frequency *r* in Equation (3).

$$\hat{r}=\{\begin{array}{cc} \frac{1}{2}\left({\hat{r}}_{F}+{\hat{r}}_{M}\right) & \text{if}{\hat{r}}_{F}\leq 0.5\text{, }{\hat{r}}_{M}\leq 0.5\left(\text{i.e. linkage phase I}\right)\\ \frac{1}{2}{\hat{r}}_{F}+\frac{1}{2}\left(1-{\hat{r}}_{M}\right) & \text{if}{\hat{r}}_{F}\leq 0.5\text{, }{\hat{r}}_{M}>0.5\;\left(\text{i.e. linkage phase II}\right)\\ \frac{1}{2}\left(1-{\hat{r}}_{F}\right)+\frac{1}{2}{\hat{r}}_{M} & \text{if}{\hat{r}}_{F}>0.5\text{, }{\hat{r}}_{M}\leq 0.5\;\left(\text{i.e. linkage phase III}\right)\\ 1\text{−}\frac{1}{2}\left({\hat{r}}_{F}+{\hat{r}}_{M}\right) & \text{if}{\hat{r}}_{F}>0.5\text{, }{\hat{r}}_{M}>0.5\;\left(\text{i.e. linkage phase IV}\right) \end{array}$$ (3)

It can be easily seen that the estimate thus defined in Equation (3) is always less than 0.5. In addition, it can be proved that the estimate in Equation (3) is also MLE of *r*, when directly calculated from its likelihood function.

### Recombination frequency estimation in scenarios 2 and 3 in clonal F_1_ progenies

In scenario 2, locus 1 has four genotypes *A*_1_*C*_1_, *A*_1_*D*_1_, *B*_1_*C*_1_, and *B*_1_*D*_1_, and locus 2 has two genotypes *X*_2_*C*_2_ and *X*_2_*D*_2_. In scenario 3, locus 1 has four genotypes *A*_1_*C*_1_, *A*_1_*D*_1_, *B*_1_*C*_1_, and *B*_1_*D*_1_, and locus 2 has two genotypes *A*_2_*X*_2_ and *B*_2_*X*_2_. Table 3 shows theoretical frequencies of the eight identifiable genotypes at the two loci. The theoretical frequencies do not contain the female recombination frequency in scenario 2, and they do not contain the male recombination frequency in scenario 3. Therefore, *r_F_* cannot be estimated in scenario 2; *r_M_* cannot be estimated in scenario 3. MLE of *r_M_* in scenario 2 can be calculated from its likelihood functions, given in Equation (4).

**Table 3:** Theoretical frequencies of the eight identifiable genotypes in the clonal F_1_ population

Genotype	Locus 1	Scenario 2 (Table 1)		Scenario 3 (Table 1)		Sample Size
		Locus 2 (*X*_2_ = *A*_2_ or *B*_2_)	Frequency	Locus 2 (*X*_2_ = *C*_2_ or *D*_2_)	Frequency	
1	*A*_1_*C*_1_	*X*_2_*C*_2_	$\frac{1}{4}(1-r_{M})$	*A*_2_*X*_2_	$\frac{1}{4}(1-r_{F})$	*n*_1_
2	*A*_1_*C*_1_	*X*_2_*D*_2_	$\frac{1}{4}r_{M}$	*B*_2_*X*_2_	$\frac{1}{4}r_{F}$	*n*_2_
3	*A*_1_*D*_1_	*X*_2_*C*_2_	$\frac{1}{4}r_{M}$	*A*_2_*X*_2_	$\frac{1}{4}(1-r_{F})$	*n*_3_
4	*A*_1_*D*_1_	*X*_2_*D*_2_	$\frac{1}{4}(1-r_{M})$	*B*_2_*X*_2_	$\frac{1}{4}r_{F}$	*n*_4_
5	*B*_1_*C*_1_	*X*_2_*C*_2_	$\frac{1}{4}(1-r_{M})$	*A*_2_*X*_2_	$\frac{1}{4}r_{F}$	*n*_5_
6	*B*_1_*C*_1_	*X*_2_*D*_2_	$\frac{1}{4}r_{M}$	*B*_2_*X*_2_	$\frac{1}{4}(1-r_{F})$	*n*_6_
7	*B*_1_*D*_1_	*X*_2_*C*_2_	$\frac{1}{4}r_{M}$	*A*_2_*X*_2_	$\frac{1}{4}r_{F}$	*n*_7_
8	*B*_1_*D*_1_	*X*_2_*D*_2_	$\frac{1}{4}(1-r_{M})$	*B*_2_*X*_2_	$\frac{1}{4}(1-r_{F})$	*n*_8_

For scenarios 2 and 3 (Table 1). *A*_1_, *B*_1_, *C*_1_, and *D*_1_ are the four alleles at locus 1. For scenario 2, *X*_2_ (=*A*_2_ or *B*_2_), *C*_2_, and *D*_2_ are the three alleles at locus 2. For scenario 3, *A*_2_, *B*_2_, and *X*_2_ (=*C*_2_ or *D*_2_) are the three alleles at locus 2. Recombination frequencies in the female and male parents are denoted as *r_F_* and *r_M_*, respectively. The last column gives the symbol of observed sample size of each genotype.

$${\hat{r}}_{M}=\frac{n_{2:3}+n_{6:7}}{n}$$, (4)

where *n_i_* is the observed sample size for the *i*th genotype (Table 3), *n_i_*_:_*_j_* is the sum of *n_i_* to *n_j_*, and *n* is the total sample size (*i.e.*, *n*=*n*_1:8_). Define the estimate of *r* in Equation (5).

$$\hat{r}=\{\begin{array}{cc} {\hat{r}}_{M} & \text{if }{\hat{r}}_{M}\leq 0.5\\ 1-{\hat{r}}_{M} & \text{otherwise} \end{array}$$. (5)

It can be easily seen that the estimate thus defined is less than 0.5. In addition, the estimate in Equation (5) is MLE of *r*, when directly calculated from its likelihood function.

MLE of *r*_*F*_ in scenario 3 can be calculated from its likelihood function, given in Equation (6).

$${\hat{r}}_{F}=\frac{n_{2}+n_{4:5}+n_{7}}{n}$$, (6)

where *n_i_* is the observed sample size of the *i*th genotype (Table 3), *n_i_*_:_*_j_* is the sum of *n_i_* to *n_j_*, and *n* is the total sample size (*i.e.*, *n*=*n*_1:8_). Define the estimate of *r* in Equation (7).

$$\hat{r}=\{\begin{array}{cc} {\hat{r}}_{F} & \text{if}{\hat{r}}_{F}\leq 0.5\\ 1-{\hat{r}}_{F} & \text{otherwise} \end{array}$$ (7)

Similar to Equation (5), the estimate thus defined is less than 0.5, and is MLE of *r*.

### Recombination frequency estimation in scenario 4 in clonal F_1_ progenies

In this scenario, locus 1 has four genotypes *A*_1_*C*_1_, *A*_1_*D*_1_, *B*_1_*C*_1_, and *B*_1_*D*_1_, and locus 2 has three genotypes *A*_2_*A*_2_, *A*_2_*B*_2_, and *B*_2_*B*_2_. Table 4 shows theoretical frequencies of the 12 identifiable genotypes at the two loci. Information on *r_F_* and *r_M_* is confounded in half of the genotypes. MLE of *r_F_* and *r_M_* using the other half of the genotypes are given in Equation (8).

**Table 4:** Theoretical frequencies of the 12 identifiable genotypes in the clonal F_1_ population

Genotype	Locus 1	Locus 2 (AB = CD)	Frequency	Combined Recombination Frequency				Sample Size
				Phase I	Phase II	Phase III	Phase IV	
1	*A*_1_*C*_1_	*A*_2_*A*_2_	$\frac{1}{4}(1-r_{F})(1-r_{M})$	$\frac{1}{4}{\left(1-r\right)}^{2}$	$\frac{1}{4}r(1-r)$	$\frac{1}{4}r(1-r)$	$\frac{1}{4}r^{2}$	*n*_1_
2	*A*_1_*C*_1_	*A*_2_*B*_2_	$\frac{1}{4}(1-r_{F})r_{M}+\frac{1}{4}r_{F}(1-r_{M})$	*$\frac{1}{2}r(1-r)$*	$\frac{1}{4}(1-2r+2r^{2})$	$\frac{1}{4}(1-2r+2r^{2})$	$\frac{1}{2}r(1-r)$	*n*_2_
3	*A*_1_*C*_1_	*B*_2_*B*_2_	$\frac{1}{4}r_{F}r_{M}$	$\frac{1}{4}r^{2}$	$\frac{1}{4}r(1-r)$	$\frac{1}{4}r(1-r)$	$\frac{1}{4}{\left(1-r\right)}^{2}$	*n*_3_
4	*A*_1_*D*_1_	*A*_2_*A*_2_	$\frac{1}{4}(1-r_{F})r_{M}$	$\frac{1}{4}r(1-r)$	$\frac{1}{4}{\left(1-r\right)}^{2}$	$\frac{1}{4}r^{2}$	$\frac{1}{4}r(1-r)$	*n*_4_
5	*A*_1_*D*_1_	*A*_2_*B*_2_	$\frac{1}{4}(1-r_{F})(1-r_{M})+\frac{1}{4}r_{F}r_{M}$	$\frac{1}{4}(1-2r+2r^{2})$	$\frac{1}{2}r(1-r)$	$\frac{1}{2}r(1-r)$	$\frac{1}{4}(1-2r+2r^{2})$	*n*_5_
6	*A*_1_*D*_1_	*B*_2_*B*_2_	$\frac{1}{4}r_{F}(1-r_{M})$	$\frac{1}{4}r(1-r)$	$\frac{1}{4}r^{2}$	$\frac{1}{4}{\left(1-r\right)}^{2}$	$\frac{1}{4}r(1-r)$	*n*_6_
7	*B*_1_*C*_1_	*A*_2_*A*_2_	$\frac{1}{4}r_{F}(1-r_{M})$	$\frac{1}{4}r(1-r)$	$\frac{1}{4}r^{2}$	$\frac{1}{4}{\left(1-r\right)}^{2}$	$\frac{1}{4}r(1-r)$	*n*_7_
8	*B*_1_*C*_1_	*A*_2_*B*_2_	$\frac{1}{4}(1-r_{F})(1-r_{M})+\frac{1}{4}r_{F}r_{M}$	$\frac{1}{4}(1-2r+2r^{2})$	$\frac{1}{2}r(1-r)$	$\frac{1}{2}r(1-r)$	$\frac{1}{4}(1-2r+2r^{2})$	*n*_8_
9	*B*_1_*C*_1_	*B*_2_*B*_2_	$\frac{1}{4}(1-r_{F})r_{M}$	$\frac{1}{4}r(1-r)$	$\frac{1}{4}{\left(1-r\right)}^{2}$	$\frac{1}{4}r^{2}$	$\frac{1}{4}r(1-r)$	*n*_9_
10	*B*_1_*D*_1_	*A*_2_*A*_2_	$\frac{1}{4}r_{F}r_{M}$	$\frac{1}{4}r^{2}$	$\frac{1}{4}r(1-r)$	$\frac{1}{4}r(1-r)$	$\frac{1}{4}{\left(1-r\right)}^{2}$	*n*_10_
11	*B*_1_*D*_1_	*A*_2_*B*_2_	$\frac{1}{4}(1-r_{F})r_{M}+\frac{1}{4}r_{F}(1-r_{M})$	$\frac{1}{2}r(1-r)$	$\frac{1}{4}(1-2r+2r^{2})$	$\frac{1}{4}(1-2r+2r^{2})$	$\frac{1}{2}r(1-r)$	*n*_11_
12	*B*_1_*D*_1_	*B*_2_*B*_2_	$\frac{1}{4}(1-r_{F})(1-r_{M})$	$\frac{1}{4}{\left(1-r\right)}^{2}$	$\frac{1}{4}r(1-r)$	$\frac{1}{4}r(1-r)$	$\frac{1}{4}r^{2}$	*n*_12_

For scenario 4 (Table 1). *A*_1_, *B*_1_, *C*_1_, and *D*_1_ are the four alleles at locus 1. *A*_2_ and *B*_2_ are the two alleles at locus 2. Recombination frequencies in the female and male parents are denoted as *r_F_* and *r_M_*, respectively. The combined recombination frequency is denoted as *r*. The last column gives the symbol of observed sample size of each genotype. For linkage phase I, female and male parents have genotypes *A*_1_*A*_2_/*B*_1_*B*_2_ and *C*_1_*A*_2_/*D*_1_*B*_2_, respectively. For linkage phase II, female and male parents have genotypes *A*_1_*A*_2_/*B*_1_*B*_2_, and *C*_1_*B*_2_/*D*_1_*A*_2_, respectively. For linkage phase III, female and male parents have genotypes *A*_1_*B*_2_/*B*_1_*A*_2_, and *C*_1_*A*_2_/*D*_1_*B*_2_, respectively. For linkage phase IV, female and male parents have genotypes *A*_1_*B*_2_/*B*_1_*A*_2_, and *C*_1_*B*_2_/*D*_1_*A*_2_, respectively.

$${\hat{r}}_{F}=\frac{n_{3}+n_{6:7}+n_{10}}{n_{1}+n_{3:4}+n_{6:7}+n_{9:10}+n_{12}},{\hat{r}}_{M}=\frac{n_{3:4}+n_{9:10}}{n_{1}+n_{3:4}+n_{6:7}+n_{9:10}+n_{12}},$$ (8)

where *n_i_* is the observed sample sizes of the *i*th genotype and *n_i_*_:_*_j_* is the sum of *n_i_* to *n_j_*.

As stated, estimated *r_F_* and *r_M_* in Equation (8) can be used in determining the linkage phases in both parents. Then, the theoretical frequencies of the 12 genotypes can be calculated based on the combined recombination frequency *r* (Table 4), from which the likelihood function can be constructed to estimate MLE of *r*. However, there is no analytic solution for MLE of *r*, and therefore some iterative algorithms have to be used (Sun *et al.* 2012). As an example, Newton-Raphson method for estimating MLE of *r* was given in Supplementary Materials (see File S1). Because the theoretical frequencies (Table 4) are calculated from the identified linkage phase, the estimated *r* is less than 0.5 when the two loci are genetically linked.

### Recombination frequency estimation in scenarios 5 and 7 in clonal F_1_ progenies

In scenario 5, locus 1 has two genotypes *X*_1_*C*_1_ and *X*_1_*D*_1_, and locus 2 has two genotypes *X*_2_*C*_2_ and *X*_2_*D*_2_. In scenario 6, locus 1 has two genotypes *A*_1_*X*_1_ and *B*_1_*X*_1_, and locus 2 has two genotypes *A*_2_*X*_2_ and *B*_2_*X*_2_. Table 5 shows theoretical frequencies of the four identifiable genotypes at the two loci. Obviously, theoretical frequencies do not contain the female recombination frequency in scenario 5 and do not contain the male recombination frequency in scenario 7. Thus, *r_F_* cannot be estimated in scenario 5; *r*_*M*_ cannot be estimated in scenario 7. MLE of *r_M_* in scenario 5 can be calculated from its likelihood functions, given in Equation (9).

**Table 5:** Theoretical frequencies of the four identifiable genotypes in the clonal F_1_ population

Genotype	Scenario 5 (Table 1)			Scenario 7 (Table 1)			Sample Size
	Locus 1 (*X*_1_= *A*_1_ or *B*_1_)	Locus 2 (*X*_2_= *A*_2_ or *B*_2_)	Frequency	Locus 1 (*X*_1_= *C*_1_ or *D*_1_)	Locus 2 (*X*_2_= *C*_2_ or *D*_2_)	Frequency	
1	*X*_1_*C*_1_	*X*_2_*C*_2_	$\frac{1}{2}(1-r_{M})$	*A*_1_*X*_1_	*A*_2_*X*_2_	$\frac{1}{2}(1-r_{F})$	*n*_1_
2	*X*_1_*C*_1_	*X*_2_*D*_2_	$\frac{1}{2}r_{M}$	*A*_1_*X*_1_	*B*_2_*X*_2_	$\frac{1}{2}r_{F}$	*n*_2_
3	*X*_1_*D*_1_	*X*_2_*C*_2_	$\frac{1}{2}r_{M}$	*B*_1_*X*_1_	*A*_2_*X*_2_	$\frac{1}{2}r_{F}$	*n*_3_
4	*X*_1_*D*_1_	*X*_2_*D*_2_	$\frac{1}{2}(1-r_{M})$	*B*_1_*X*_1_	*B*_2_*X*_2_	$\frac{1}{2}(1-r_{F})$	*n*_4_

For scenarios 5 and 7 (Table 1). For scenario 5, *X*_1_ (=*A*_1_ or *B*_1_), *C*_1_ and *D*_1_ are the three alleles at locus 1; *X*_2_ (=*A*_2_ or *B*_2_), *C*_2_, and *D*_2_ are the three alleles at locus 2. For scenario 7, *A*_1_, *B*_1_, and *X*_1_ (=*C*_1_ or *D*_1_) are the three alleles at locus 1; *A*_2_, *B*_2_, and *X*_2_ (=*C*_2_ or *D*_2_) are the three alleles at locus 2. Recombination frequencies in the female and male parents are denoted as *r_F_* and *r_M_*, respectively. The last column gives the symbol of observed sample size of each genotype.

$${\hat{r}}_{M}=\frac{n_{2:3}}{n}$$, (9)

where *n_i_* is the observed sample size of the *i*th genotype (Table 5), *n_i_*_:_*_j_* is the sum of *n_i_* to *n_j_*, and *n* is the total sample size (*i.e.*, *n*=*n*_1:4_). Define the estimate of *r* in Equation (10).

$$\hat{r}=\{\begin{array}{cc} {\hat{r}}_{M} & \text{if}{\hat{r}}_{M}\leq 0.5\\ 1-{\hat{r}}_{M} & \text{otherwise} \end{array}$$ (10)

MLE of *r_F_* in scenario 7 can be calculated from its likelihood functions, given in Equation (11). Define the estimate of *r* in Equation (12).

$${\hat{r}}_{F}=\frac{n_{2:3}}{n}$$ (11)

$$\hat{r}=\{\begin{array}{cc} {\hat{r}}_{F} & \text{if}{\hat{r}}_{F}\leq 0.5\\ 1-{\hat{r}}_{F} & \text{otherwise} \end{array}$$ (12)

Similar to Equation (5) and Equation (7), the estimates defined in Equation (10) and Equation (12) are less than 0.5, and are MLE of *r* for scenarios 5 and 7, respectively.

### Recombination frequency estimation in scenarios 6 and 8 in clonal F_1_ progenies

In scenario 6, locus 1 has two genotypes *X*_1_*C*_1_ and *X*_1_*D*_1_, and locus 2 has three genotypes *A*_2_*A*_2_, *A*_2_*B*_2_, and *B*_2_*B*_2_. In scenario 8, locus 1 has two genotypes *A*_1_*X*_1_ and *B*_1_*X*_1_, and locus 2 has three genotypes *A*_2_*A*_2_, *A*_2_*B*_2_, and *B*_2_*B*_2_. Table 6 shows theoretical frequencies of the six identifiable genotypes at the two linked loci. The theoretical frequencies do not contain the female recombination frequency in scenario 6 and do not contain the male recombination frequency in scenario 8. Thus, *r_F_* cannot be estimated in scenario 6, and *r_M_* cannot be estimated in scenario 8. MLE of *r_M_* in scenario 6 can be calculated from its likelihood function, given in Equation (13).

**Table 6:** Theoretical frequencies of the six identifiable genotypes in the clonal F_1_ population

Genotype	Locus 1		Locus 2	Frequency		Sample Size
	Scenario 6	Scenario 8		Scenario 6	Scenario 8	
1	*X*_1_*C*_1_	*A*_1_*X*_1_	*A*_2_*A*_2_	$\frac{1}{4}(1-r_{M})$	$\frac{1}{4}(1-r_{F})$	*n*_1_
2	*X*_1_*C*_1_	*A*_1_*X*_1_	*A*_2_*B*_2_	$\frac{1}{4}$	$\frac{1}{4}$	*n*_2_
3	*X*_1_*C*_1_	*A*_1_*X*_1_	*B*_2_*B*_2_	$\frac{1}{4}r_{M}$	$\frac{1}{4}r_{F}$	*n*_3_
4	*X*_1_*D*_1_	*B*_1_*X*_1_	*A*_2_*A*_2_	$\frac{1}{4}r_{M}$	$\frac{1}{4}r_{F}$	*n*_4_
5	*X*_1_*D*_1_	*B*_1_*X*_1_	*A*_2_*B*_2_	$\frac{1}{4}$	$\frac{1}{4}$	*n*_5_
6	*X*_1_*D*_1_	*B*_1_*X*_1_	*B*_2_*B*_2_	$\frac{1}{4}(1-r_{M})$	$\frac{1}{4}(1-r_{F})$	*n*_6_

For scenarios 6 and 8 (Table 1). For scenario 6, *X*_1_ (=*A*_1_ or *B*_1_), *C*_1_, and *D*_1_ are the three alleles at locus 1; *A*_2_ and *B*_2_ are the two alleles at locus 2. For scenario 8, *A*_1_, *B*_1_, and *X*_1_ (=*C*_1_ or *D*_1_) are the three alleles at locus 1; *A*_2_ and *B*_2_ are the two alleles at locus 2. Recombination frequencies in the female and male parents are denoted as *r_F_* and *r_M_*, respectively. The last column gives the symbol of observed sample size of each genotype

$${\hat{r}}_{M}=\frac{n_{3:4}}{n_{1}+n_{3:4}+n_{6}}$$ (13)

where *n_i_* is the observed sample size of the *i*th genotype (Table 6) and *n_i_*_:_*_j_* is the sum of *n_i_* to *n_j_*. Define the estimate of *r* in Equation (14).

$$\hat{r}=\{\begin{array}{cc} {\hat{r}}_{M} & \text{if}{\hat{r}}_{M}\leq 0.5\\ 1-{\hat{r}}_{M} & \text{otherwise} \end{array}$$. (14)

Maximum likelihood estimates of *r_F_* in scenario 8 can be calculated from its likelihood function, given in Equation (15). Define the estimate of *r* in Equation (16).

$${\hat{r}}_{F}=\frac{n_{3:4}}{n_{1}+n_{3:4}+n_{6}}$$ (15)

$$\hat{r}=\{\begin{array}{cc} {\hat{r}}_{F} & \text{if}{\hat{r}}_{F}\leq 0.5\\ 1-{\hat{r}}_{F} & \text{otherwise} \end{array}$$ (16)

Similar to Equation (5), Equation (7), Equation (10), and Equation (12), the estimates defined in Equation (14), Equation (15), and Equation (16) are less than 0.5 and are MLE of *r* for scenarios 6 and 8, respectively.

### Recombination frequency estimation in scenario 9 in clonal F_1_ progenies

In this scenario, locus 1 has three genotypes *A*_1_*A*_1_, *A*_1_*B*_1_ and *B*_1_*B*_1_, and locus 2 has three genotypes *A*_2_*A*_2_, *A*_2_*B*_2_ and *B*_2_*B*_2_. Linkage information in both parents cannot be separated; therefore, *r_F_* and *r_M_* cannot be estimated. Linkage phases in parents are unknown before estimating the combined recombination frequency *r*. Table 7 shows theoretical frequencies of the nine identifiable genotypes at the two loci in the four potential linkage phases I to IV. For linkage phase I, female and male parents have the same genotype *A*_1_*A*_2_/*B*_1_*B*_2_. For linkage phase II, female and male parents have genotypes *A*_1_*A*_2_/*B*_1_*B*_2_ and *A*_1_*B*_2_/*B*_1_*A*_2_, respectively. For linkage phase III, female and male parents have genotypes *A*_1_*B*_2_/*B*_1_*A*_2_ and *A*_1_*A*_2_/*B*_1_*B*_2_, respectively. For linkage phase IV, female and male parents have the same genotype *A*_1_*B*_2_/*B*_1_*A*_2_. Phases II and III are equivalent in genetics and have the same genotypic frequencies.

**Table 7:** Theoretical frequencies of the nine identifiable genotypes in the clonal F_1_ population

Genotype	Locus 1	Locus 2	Expected Frequency			Sample Size
			Phase I	Phases II and III	Phase IV	
1	*A*_1_*A*_1_	*A*_2_*A*_2_	$\frac{1}{4}{\left(1-r\right)}^{2}$	$\frac{1}{4}r(1-r)$	$\frac{1}{4}r^{2}$	*n*_1_
2	*A*_1_*A*_1_	*A*_2_*B*_2_	$\frac{1}{2}r(1-r)$	$\frac{1}{4}(1-2r+2r^{2})$	$\frac{1}{2}r(1-r)$	*n*_2_
3	*A*_1_*A*_1_	*B*_2_*B*_2_	$\frac{1}{4}r^{2}$	$\frac{1}{4}r(1-r)$	$\frac{1}{4}{\left(1-r\right)}^{2}$	*n*_3_
4	*A*_1_*B*_1_	*A*_2_*A*_2_	$\frac{1}{2}r(1-r)$	$\frac{1}{4}(1-2r+2r^{2})$	$\frac{1}{2}r(1-r)$	*n*_4_
5	*A*_1_*B*_1_	*A*_2_*B*_2_	$\frac{1}{2}(1-2r+2r^{2})$	r(1−r)	$\frac{1}{2}(1-2r+2r^{2})$	*n*_5_
6	*A*_1_*B*_1_	*B*_2_*B*_2_	$\frac{1}{2}r(1-r)$	$\frac{1}{4}(1-2r+2r^{2})$	$\frac{1}{2}r(1-r)$	*n*_6_
7	*B*_1_*B*_1_	*A*_2_*A*_2_	$\frac{1}{4}r^{2}$	$\frac{1}{4}r(1-r)$	$\frac{1}{4}{\left(1-r\right)}^{2}$	*n*_7_
8	*B*_1_*B*_1_	*A*_2_*B*_2_	$\frac{1}{2}r(1-r)$	$\frac{1}{4}(1-2r+2r^{2})$	$\frac{1}{2}r(1-r)$	*n*_8_
9	*B*_1_*B*_1_	*B*_2_*B*_2_	$\frac{1}{4}{\left(1-r\right)}^{2}$	$\frac{1}{4}r(1-r)$	$\frac{1}{4}r^{2}$	*n*_9_

For scenario 9 (Table 1). *A*_1_ and *B*_1_ are the two alleles at locus 1; *A*_2_ and *B*_2_ are the two alleles at locus 2. The combined recombination frequency is denoted as *r*. The last column gives the symbol of observed sample size of each genotype. For linkage phase I, female and male parents have the same genotype *A*_1_*A*_2_/*B*_1_*B*_2_. For linkage phase II, female and male parents have genotypes *A*_1_*A*_2_/*B*_1_*B*_2_ and *A*_1_*B*_2_/*B*_1_*A*_2_, respectively. For linkage phase III, female and male parents have genotypes *A*_1_*B*_2_/*B*_1_*A*_2_ and *A*_1_*A*_2_/*B*_1_*B*_2_, respectively. For linkage phase IV, female and male parents have the same genotype *A*_1_*B*_2_/*B*_1_*A*_2_.

For linkage phases I and IV, Newton-Raphson algorithms to estimate *r* can be found in Supplementary Materials (see File S2). For linkage phases II and III, MLE of *r* can be found from Equation (17).

$$\hat{r}=\frac{1}{2}(1-\sqrt{1-2\left(n_{1}+n_{3}+n_{5}+n_{7}+n_{9}\right)/n})$$ (17)

where *n_i_* is the observed sample size of the *i*th genotype and *n* is the total sample size (*i.e.*, *n*=*n*_1:9_).

**Figure 3:**
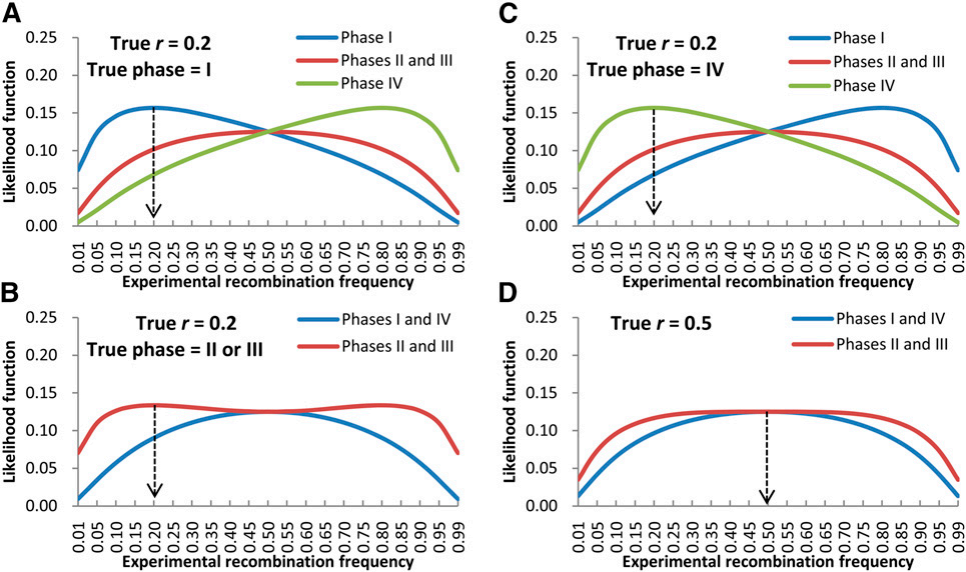
Likelihood function of experimental recombination frequency when both loci are marker category IV, *i.e.*, *A*_1_*B*_1_= *C*_1_*D*_1_ and *A*_2_*B*_2_= *C*_2_*D*_2_ (scenario 9 in Table 1), and the true recombination frequency is 0.2 (A, B, and C for close linkage) or 0.5 (D for no linkage). In this scenario, the female parent may have either genotype *A*_1_*A*_2_/*B*_1_*B*_2_ or genotype *A*_1_*B*_2_/*B*_1_*A*_2_, and so does the male parent. Therefore, there are four possible linkage phases, but only one could be true. The four possible phases are (1) the female and male parents have the same genotype *A*_1_*A*_2_/*B*_1_*B*_2_; (2) the female parent has genotype *A*_1_*A*_2_/*B*_1_*B*_2_ and the male parent has genotype *A*_1_*B*_2_/*B*_1_*A*_2_; (3) the female parent has genotype *A*_1_*B*_2_/*B*_1_*A*_2_ and the male parent has genotype *A*_1_*A*_2_/*B*_1_*B*_2_; and (4) the female and male parents have the same genotype *A*_1_*B*_2_/*B*_1_*A*_2_.

To explain how the linkage phase can be determined by the estimated *r* from the four potential linkage phases, Figure 3 shows likelihood function profiles on experimental recombination frequency when both marker loci are category IV. When true recombination frequency was 0.2 (*i.e.*, two loci were linked) and true linkage phase was I (Figure 3A), *r* was estimated at 0.2 in linkage phase I, at 0.5 in linkage phases II and III, and at 0.8 in linkage phase IV. If the true linkage phase was II or III (Figure 3B), then *r* was estimated at 0.5 in linkage phases I and IV and at 0.2 or 0.8 in linkage phases II and III. If the true linkage phase was IV (Figure 3C), then *r* was estimated at 0.8 in linkage phase I, at 0.5 in linkage phases II and III, and at 0.2 in linkage phase IV. Obviously, if the experimental phase coincides with the true linkage phase, then the estimated *r* would be the lowest among all estimates of the four potential phases, which is actually equal to its true value. In other words, the experimental phase that has the lowest estimate of *r* can be viewed as the true linkage phase, and the lowest estimate can be viewed as the true value of *r*. When estimated *r* is lowest in linkage phases II and III, the two loci are randomly assigned to phase II or phase III. If the two loci were not linked (*i.e.*, true recombination frequency is 0.5), then *r* should be estimated at approximately 0.5 in all linkage phases (Figure 3D). In this case, linkage phase does not make any sense and is randomly assigned to one of the four phases.

Consistent with previous scenarios, *r_F_* and *r_M_* need to be defined to reflect the identified linkage phase after *r* and linkage phase are determined. For this purpose, *r_F_* and *r_M_* are both assigned to *r* in linkage phase I, assigned to *r* and 1−*r*, respectively, in linkage phase II, assigned to 1−*r* and *r*, respectively, in linkage phase III, and assigned to 1−*r* in linkage phase IV. For convenience, estimates of *r_F_* and *r_M_* are given in Equation 18.

$${\hat{r}}_{F}=\{\begin{array}{cc} \hat{r} & \text{for}\;\text{phase}\;\text{I}\;\text{or}\;\text{II}\\ 1-\hat{r} & \text{for}\;\text{phase}\;\text{III}\;\text{or}\;\text{IV} \end{array},{\hat{r}}_{M}=\{\begin{array}{cc} \hat{r} & \text{for}\;\text{phase}\;\text{I}\;\text{or}\;\text{III}\\ 1-\hat{r} & \text{for}\;\text{phase}\;\text{II}\;\text{or}\;\text{IV} \end{array}.$$ (18)

### Haploid building in clonal parents from their segregating progenies

For the clonal F_1_ progenies, genotype of the female parent can be either *A*_1_*B*_1_/*A*_2_*B*_2_ or *A*_1_*B*_2_/*B*_1_*A*_2_; genotype of the male parent can be either *C*_1_*D*_1_/*C*_2_*D*_2_ or *C*_1_*D*_2_/*D*_1_*C*_2_. The linkage phase can be identified from the estimated recombination frequencies and constructed linkage maps and, finally, the four haploids in the two clonal parents can be built. Two haploids of the female parent are called HapA and HapB; those of the male parent are called HapC and HapD. Female haploid building tries to assign the two alleles *A* and *B* at each locus on the female map to haploids HapA and HapB. Male haploid building tries to assign the two alleles *C* and *D* at each locus on the male map to haploids HapC and HapD. Haploid building of ordered markers on one chromosome is similar for both parents. We use the female parent as an example to explain the building procedure.

Step 1: At the first ordered locus, allele *A* is assigned to HapA; allele *B* is assigned to HapB.
Step 2: For the second ordered locus, if estimated *r_F_* with the first locus is lower than 0.5, then allele *A* is assigned to HapA; allele *B* is assigned to HapB. Otherwise, allele *B* is assigned to HapA and allele *A* is assigned to HapB.
Step 3: For the next ordered locus, if estimated *r_F_* with its previous locus is lower than 0.5, then allele *A* is assigned to HapA, and allele *B* is assigned to HapB if allele *A* at the previous locus is on HapA; allele *B* is assigned to HapA and allele *A* is assigned to HapB if allele *B* at the previous locus is on HapA. If estimated *r_F_* with its previous locus is more than 0.5, then allele *B* is assigned to HapA, and allele *A* is assigned to HapB if allele *A* at the previous locus is on HapA; allele *A* is assigned to HapA and allele *B* is assigned to HapB if allele *B* at the previous locus is on HapA.
Step 4: Repeat the process from step 3 until the last ordered locus on the chromosome.

### Marker categories and linkage analysis in double cross populations

Double cross populations in plants have four inbred lines, A, B, C, and D, as parents that are homozygous at most chromosomal locations (Figure S1). First, one F_1_ hybrid is made between inbred lines A and B; the other F_1_ hybrid is made between inbred lines C and D. Then, a double cross is made between the two F_1_ hybrids; one is used as female and the other one is used as male. When polymorphism markers are screened in the four inbred lines, the four alleles in double cross populations can be clearly assigned. In this case, five marker categories can be differentiated on the number of identifiable alleles in the four original lines and the number of identifiable genotypes in their double cross progenies (Figure S2). Categories I to III are similar to those in clonal F_1_. Category IV in clonal F_1_ can be further divided into two categories in double cross, which are denoted as categories IV and V. For category IV (or A = CB = D), allele *A* is the same as allele *C*, and allele *B* is the same as allele *D*. For category V (or A = DB = C), allele *A* is the same as allele *D*, and allele *B* is the same as allele *C*.

For two loci, genotypes of the four inbred lines are *A*_1_*A*_1_, *B*_1_*B*_1_, *C*_1_*C*_1_, and *D*_1_*D*_1_ at locus 1, and *A*_2_*A*_2_, *B*_2_*B*_2_, *C*_2_*C*_2_, and *D*_2_*D*_2_ at locus 2. Linkage phases in the female and male F_1_ hybrids are known as *A*_1_*A*_2_/*B*_1_*B*_2_ and *C*_1_*C*_2_/*D*_1_*D*_2_, which are equivalent to linkage phase I in clonal F_1_. When category V is absent, scenarios 1 to 9 in clonal F_1_ are still applicable in double cross populations. For these scenarios, theoretical genotypic frequencies and formulas in estimating *r_F_*, *r_M_*, and *r* are the same as those for clonal F_1_ in the case of linkage phase I, *i.e.*, *r_F_* and *r_M_* are both smaller than 0.5 if they can be estimated.

There are five new scenarios for recombination frequency estimation when category V is present. In scenario 10, locus 1 is category I and locus 2 is category V. In scenario 11, locus 1 is category II and locus 2 is category V. In scenario 12, locus 1 is category III and locus 2 is category V. In scenario 13, locus 1 is category IV and locus 2 is category V. In scenario 14, the two loci are category V.

In scenario 10, the 12 identifiable genotypes are the same as scenario 4 in Table 4. Theoretical frequency of each genotype is equal to the corresponding value in Table 4 by substituting *r_M_* with 1−*r_M_* (see Table S2). In scenario 11, the six identifiable genotypes are the same as scenario 6 in Table 6. Theoretical frequency of each genotype is equal to the corresponding value of scenario 6 in Table 6 by substituting *r_M_* with 1−*r_M_* (Table S3). In scenario 12, the six identifiable genotypes and their theoretical frequencies are the same as scenario 8 in Table 6 (Table S3). In scenario 13, genotypes and their theoretical frequencies are the same as linkage phases II and III of scenario 9 in Table 7 (Table S4). In scenario 14, genotypes and their theoretical frequencies are the same as linkage phase I of scenario 9 in Table 7 (Table S4). Methods for estimating *r* are similar to the corresponding scenarios in clonal F_1_. For convenience, theoretical genotypic frequencies at two loci for scenarios 10 to 14 are given in Table S2, Table S3, and Table S4.

### LOD score in testing the linkage relationship between two loci

The existence of the linkage can be tested by the following two hypotheses.

$$H_{0}:r=0.5\;\text{vs.}\;H_{A}:r<0\mathrm{.5,}$$

where *H*_0_ is the null hypothesis corresponding to no genetic linkage, *H_A_* is the alternative hypothesis corresponding to the linkage relationship between two loci, and *r* is the combined recombination frequency. The log-likelihood function under the null hypothesis is $\log\;L_{0}=\log\;L(r=0.5)$, whereas the log-likelihood function under the alternative hypothesis is $\log\;L_{A}=\log\;L(r=\hat{r})$. The *LOD* score can be calculated from the log-likelihoods under the two hypotheses, *i.e.*, $LOD=\log\;L_{A}-\log\;L_{0}$, where log is the logarithm function of base 10.

### One simulated population and one actual population

We considered one chromosome with 20 evenly distributed markers in simulation. Recombination frequencies between any two neighboring markers were set at 0.05, equivalent to a genetic distance of 5.27 cM using Haldane mapping function (Haldane 1919).

One population with 200 clonal F_1_ progenies was simulated by the genetics and breeding simulation tool of QuLine (Wang *et al.* 2003, 2004). Five markers were randomly chosen and assigned to each of the four categories (Figure 2). Markers 8, 11, 14, 17, and 19 were category I; markers 1, 2, 13, 15, and 20 were assigned to category II; markers 4, 5, 7, 9, and 18 were assigned to category III. Alleles A=C≠B=D for markers 10 and 12, and alleles A=D≠B=C for markers 3, 6 and 16, with both representing markers of category IV. To simulate the unknown linkage phases, alleles *A* and *B* were purposely swapped for markers 5 and 18. Alleles *C* and *D* were swapped for markers 14, 15, and 20. For markers 8, 12, and 16, alleles *A* and *B* were swapped and alleles *C* and *D* were swapped.

The actual double cross population used in this study was derived from four maize inbred lines, developed by the College of Agronomy, Henan Agricultural University (Li *et al.* 2013). The population consists of 277 double cross individuals. Two single crosses were first made in Zhengzhou, Henan, China, in summer 2008. One was between maize inbred lines 276 and 72, and the other was between maize inbred lines A188 and Jiao51. The two single crosses were then planted in Ledong, Hainan, China, in winter 2008, and the double cross was made at the flowering stage. The double cross population was planted in Zhengzhou in spring 2009 for phenotyping. Polymorphism of SSR molecular markers was first screened in the two single crosses. Then, the double cross population was genotyped by 220 polymorphism SSR markers. The original four parental lines were not genotyped. Therefore, linkage phases in this population are unknown, and the linkage analysis method of clonal F_1_ is applicable.

A threshold of recombination frequency 0.3 was used for marker grouping in the actual population. A combined algorithm of nearest neighbor and Two-opt algorithm of Traveling Salesman Problem (Lin and Kernighan 1973) was used for marker ordering in both populations. The nearest neighbor algorithm was used to determine an initial solution that quickly yielded a short tour, but usually not the shortest one. Then Two-opt algorithm was used for improving the solution (Supplementary Materials, see File S3). Algorithms for estimating recombination frequencies and building linkage map were implemented in the software called GACD (available from www.isbreeding.net). For comparison, JoinMap4.1, OneMap, and R/qtl were used for linkage map construction in the simulated population. The mapping algorithm in JoinMap4.1 was maximum likelihood mapping with the following parameters: chain length = 1000; initial acceptance probability = 0.25; cooling control parameter = 0.001; and stop after 10000 chins without improvement. Function “order.seq” in OneMap was used for ordering, where the best order was determined in a window size of five markers. The best order in R/qtl was determined by function “orderMarker,” where the initial order was established by a greedy algorithm and was refined by rippling. In the simulated population, Haldane mapping function was used to convert recombination frequency (*r*) to map distance (*d*) in cM. In the maize population, Kosambi mapping function (Kosambi 1944) was used to convert *r* to *d* in cM.

## Results

### Estimated recombination frequencies in simulated population

Theoretical recombination frequencies between the 20 simulated markers were shown in the upper triangular matrix (Table S5). The closer to the diagonal, the lower the recombination frequencies would be. For example, recombination frequencies between marker 1 and markers 2, 8, and 19 were 0.05, 0.26, and 0.42, respectively. Recombination frequencies of marker pairs 8 and 9, 8 and 15, and 8 and 20 were 0.05, 0.26, and 0.36 (Table S5), respectively.

The lower triangular matrix of Table S5 showed the estimated recombination frequencies between the 20 markers. Combined recombination frequencies cannot be estimated if one marker is category II and the other one is category III. For example, recombination frequencies between marker pair 1 and 4 and marker pair 5 and 13 cannot be estimated, which were left as blank in Table S5. When the combined recombination frequencies could be estimated, the estimates were close to their true values. For example, marker 1 was category II, its true recombination frequencies with markers 2, 8, and 19 were 0.05, 0.26, and 0.42, and the estimates were 0.05, 0.22, and 0.48, respectively. Marker 8 was category I, its true recombination frequencies with markers 9, 15, and 20 were 0.05, 0.26, and 0.36, and the estimates were 0.03, 0.27, and 0.42, respectively.

If combined recombination frequency cannot be estimated, then the corresponding marker distance and LOD score cannot be calculated either. The upper triangular matrix showed the estimated map distance between the 20 markers (Table S6). The closer between two markers, the smaller the estimated distance is. For example, the true recombination frequencies of marker pairs 1 and 2, 1 and 8, and 1 and 19 were 0.05, 0.26, and 0.42 (Table S5). Their estimated distances were 5.3 cM, 29.0 cM, and 160.9 cM (Table S6), respectively. The true recombination frequencies of marker pairs 8 and 9, 8 and 15, and 8 and 20 were 0.05, 0.26, and 0.36 (Table S5). Their estimated distances were 3.1 cM, 37.8 cM, and 88.6 cM (Table S6), respectively. It should be noted that the map length of a chromosome is calculated from lengths of individual ordered intervals, rather than the recombination frequency between the first and the last markers.

The lower triangular matrix of Table S6 showed LOD score between the 20 markers. The closer between two markers, the greater the LOD score is. For example, the true recombination frequencies between marker pairs 1 and 2, 1 and 8, and 1 and 19 were 0.05, 0.26, and 0.42 (Table S5). Their LOD scores were 43.0, 14.4, and 0.1 (Table S6), respectively. The true recombination frequencies between marker pairs 8 and 9, 8 and 15, and 8 and 20 were 0.05, 0.26, and 0.36 (Table S5). Their LOD scores were 48.5, 10.0, and 1.3 (Table S6), respectively.

### Marker ordering in simulated population

**Figure 4:**
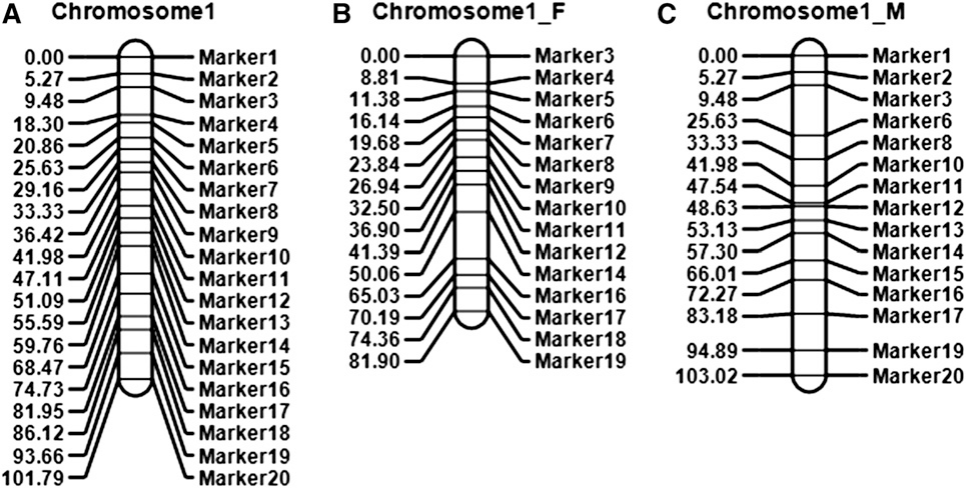
The combined, female, and male linkage maps of 20 markers in a simulated clonal F_1_ population with 200 progenies. Haldane mapping function was used to convert recombination frequency to genetic distance.

Estimates of the combined recombination frequencies were used to order the 20 markers, and the best order with the shortest map length was shown in Figure 4A, which was the same as the predefined order. The estimated length of the chromosome was 101.79 cM, close to the true length 100.13 cM. Average marker distance was 5.36 cM, close to the true value 5.27 cM.

The female map does not contain markers of category II, and the male map does not contain markers of category III. The order of markers in the female and male maps were the same as that in the combined map, but map distances between markers were estimated by the female and male recombination frequencies, respectively. In the simulated population, lengths of the female and male maps were 81.90 cM and 103.02 cM, respectively (Figure 4, B and C). For the 20 markers, 1, 2, 13, 15, and 20 are category II (Table S4 and Table S5) and therefore do not appear on the female map. Marker 3 was located at the beginning and marker 19 located at the end on the female map, which explained the reduced female map length. Markers, 4, 5, 7, 9, and 17 are category III (Table S4 and Table S5); therefore, they do not appear on the male map. However, marker 1 was still located at the beginning and 20 was still located at the end on the male map, which explained the map length similar to the combined one.

### Four haploids of two parents in the simulated population

Using estimated *r_F_* and *r_M_* between neighboring markers, four haploids of parents at 20 marker loci were determined (Table 8). The first marker is category II, which had no polymorphism in the female parent. It was not included on the female map, but it was included on the male map (Figure 4, B and C). Alleles on HapA and HapB were represented by *X*, which can be either allele *A* or allele *B*. Alleles on HapC and HapD were *C* and *D*, respectively. The second marker is category II as well. The estimated *r_M_* with previous marker was 0.05 (less than 0.5). Alleles on HapA and HapB were represented by *X*, which could be either allele *A* or allele *B*. Alleles on HapC and HapD were *C* and *D*, respectively, which were the same haploids as those of the previous locus. Marker 3 was the first on the female map (Figure 4B). Alleles *A* and *B* were on HapA and HapB (Table 8). It was the third marker on the male map (Figure 4C). Estimated *r_M_* with previous marker was 0.975, which was more than 0.5. Alleles *D* and *C* were assigned to HapC and HapD, respectively, which were opposite to the previous locus. The four haploids in Table 8 were consistent with the predefined haploid types.

**Table 8:** Haploid building of female and male parents and the updated marker category

Marker	Category	Female Parent		Male Parent		Updated Category
		HapA	HapB	HapC	HapD	
1	II	*X*	*X*	*C*	*D*	II
2	II	*X*	*X*	*C*	*D*	II
3	IV	*A*	*B*	*D*	*C*	V
4	III	*A*	*B*	*X*	*X*	III
5	III	*B*	*A*	*X*	*X*	III
6	IV	*A*	*B*	*D*	*C*	V
7	III	*A*	*B*	*X*	*X*	III
8	I	*B*	*A*	*D*	*C*	I
9	III	*A*	*B*	*X*	*X*	III
10	IV	*A*	*B*	*C*	*D*	IV
11	I	*A*	*B*	*C*	*D*	I
12	IV	*B*	*A*	*D*	*C*	IV
13	II	*X*	*X*	*C*	*D*	II
14	I	*A*	*B*	*D*	*C*	I
15	II	*X*	*X*	*D*	*C*	II
16	IV	*B*	*A*	*C*	*D*	V
17	I	*A*	*B*	*C*	*D*	I
18	III	*B*	*A*	*X*	*X*	III
19	I	*A*	*B*	*C*	*D*	I
20	II	*X*	*X*	*D*	*C*	II

HapA and HapB are the two haploids of the female parent. HapC and HapD are the two haploids of the male parent.

Marker category IV in clonal F_1_ can be further divided into two categories, *i.e.*, categories IV and V in double cross (Figure S2). In a simulated population, markers 3, 6, 10, 12, and 16 were category IV. Taking marker 3 as an example, alleles on HapA, HapB, HapC, and HapD were *A*, *B*, *D*, and *C*, respectively. Its category was redefined as category V of double cross (Table 8).

For HapA and HapB of the female parents (Table 8), if we exchange alleles *A* and *B* at loci 5, 8, 12, 16, and 18, then HapA will have *A* alleles at all loci and HapB will have *B* alleles at all loci. For HapC and HapD of the male parents (Table 8), if we exchange alleles *C* and *D* at loci 3, 6, 8, 12, 14, 15, and 20, then HapC will have *C* alleles at all loci and HapD will have *D* alleles at all loci. If the four haploids built earlier could be viewed as haploids of the four inbred lines in a double cross, then clonal F_1_ is equivalent to double cross!

### Comparison with JoinMap, OneMap, and R/qtl for linkage map construction

General information of combined linkage maps of the simulated population built by GACD, JoinMap4.1, OneMap, and R/qtl were shown (Table S7). R/qtl can only conduct linkage mapping in phase-known double cross, so marker categories and genotypes after haploid building were imported into R/qtl. Marker orders given by GACD, OneMap, and R/qtl were the same as the predefined order in the simulated model. However, marker order given by JoinMap4.1 was far from the predefined (Table S7). The first and last markers were Marker 12 and Marker 18, respectively. The true map length was 100.13 cM. Length was estimated at 101.79 cM from GACD, 15211.04 cM from JoinMap, 103.83 cM from OneMap, or 104.22 cM from R/qtl. The reason for the extremely large map length from JoinMap was the estimated value of 0.5 of recombination frequency between some neighboring markers in the female or male maps, which was converted to a distance of 10,000.0 cM in JoinMap. For example, recombination frequency between markers 3 and 5 belonging to category V and III was estimated at 0.5 on the female map, corresponding to a distance of 10,000.0 cM on the female map and 5007.99 cM on the combined map. Time spent for building the maps was 8 sec by GACD, 30 sec by JoinMap, 455 sec by OneMap, and 63 sec by R/qtl on a computer with 1.60 GHz CPU and 3.00 GB RAM.

Comparison of different software packages was also conducted in a simulated clonal F_1_ population with distorted markers (Supplementary Materials, see File S4) and a simulated clonal F_1_ population with 200 individuals and 200 markers belonging to category IV (Supplementary Materials, see File S5). A greater advantage was observed for the marker number 200 in one single chromosome (Table S8). GACD took 0.5 min, JoinMAP took 5 min, OneMAP took 537 min, and R did not output any results. GACD results in the shortest linkage map closest to the true length in the shortest time (Table S8). The reason may be as follows. Previous studies tried to estimate recombination frequency, determine linkage phase, and build linkage map simultaneously. In our study, we first estimate all pair-wise recombination frequencies (*i.e.*, step 1). Linkage phases were determined from the estimated recombination frequencies (*i.e.*, step 2). Linkage map was built based on the matrix of all pair-wise recombination frequencies (*i.e.*, step 3). Finally, the four haploids were built from the completed linkage maps (*i.e.*, step 4). Separating a complicated genetic question into four clearly defined steps results in more accurate genetic linkage maps in shorter time. In addition, we believe the adoption of the optimization algorithm in solving the Traveling Salesman Problems also contributes to the ordering efficiency.

### Linkage maps in actual double cross population

In the actual population, the missing marker rate was at 6.49%. Among the 220 markers, 60 markers showed segregation distortion under significance level 0.05. Recombination frequencies of all marker pairs were estimated and then used for linkage map construction. The combined genetic linkage map was constructed by 219 SSR molecular markers using the software GACD. One marker cannot be linked with any other markers and was deleted. The 10 chromosomes had 25, 28, 25, 24, 21, 19, 18, 16, 25, and 18 relatively evenly distributed markers, respectively (Figure S3). The whole genome was 1778.09 cM in length, and the average marker distance was 8.51 cM.

The 10 female chromosomes (Figure S3) had 19, 19, 20, 13, 16, 13, 12, 14, 17, and 15 markers, respectively, with a total of 158 markers. The total female map length was 1796.92 cM. The 10 male chromosomes (Figure S3) had 18, 19, 22, 21, 17, 14, 14, 9, 19, and 15 markers, respectively, with a total of 168 markers. The total male map length was 1599.13 cM.

Li *et al.* (2013) used JoinMap4.0 to build the linkage maps for this actual population. Kosambi mapping function was used to convert recombination frequency to genetic distance. As indicated in their study, 213 makers were included in the 11 linkage groups of the combined map. The other seven markers were not linked. The whole genome was 1626.3 cM, and the average marker distance was 1626.3/(213−11) = 8.05 cM. Compared with the map by JoinMap, our method provided a methodology that has the following advantages. First, the number of linkage groups from GACD was the same as the number of chromosomes in maize genome. Second, GACD links more markers than JoinMap. One marker was identified by GACD to be unlinked, but seven markers were unlinked by JoinMap. The length of genome from GACD was slightly longer than that from JoinMap. This may be caused by two possible reasons: more markers were included on the linkage maps by GACD and chromosome 2 was split into two by JoinMap.

## Discussion

### Linkage analysis in clonal F_1_ progenies using all informative markers

Linkage analysis and map construction are crucial steps in genetic studies of quantitative traits and provide the basis for map-based gene cloning and marker-assisted breeding. A key to linkage map construction is the accurate estimation of recombination frequency, which has been widely studied for various populations in plants over a long period of time (Fisher 1935; Haldane and Smith 1947; Morton 1955; Smith 1959; Bailey 1961; Ott 1974; Nordheim *et al.* 1983; Ritter *et al.* 1990, 1996; Wu *et al.* 2002a, b; van Ooijen 2011). Säll and Nilsson (1994) showed that the accuracy of recombination frequency estimation was affected by limited sample size, heterogeneity in recombination frequency between sexes or among meiosis, and factors that distort the segregation misclassification or differential viability. Hackett and Broadfoot (2003) investigated that accuracy of linkage maps was reduced by missing values and/or typing errors in genotyping, but segregation distortion had little effect on marker order. Sun *et al.* (2012) investigated the estimation efficiency of recombination frequency in 12 bi-parental populations. They concluded that larger population size and smaller recombination frequency resulted in higher LOD score and smaller deviation. Advanced backcrossing and selfing populations had lower precision in estimating the recombination frequency due to the enlarged recombination frequency.

The four marker categories (Figure 2) considered in this study represented all polymorphism markers that could provide the required information for genetic studies. Linkage analysis was conducted for markers not only in the same category but also in different categories. Three sets of recombination frequencies could be estimated accordingly to build the female, male, and combined linkage maps simultaneously. Results from simulated populations and one actual maize population demonstrated the accuracy of the proposed method and its advantages over other software packages. Methodology developed in this study, together with the freely available GACD software, provides an integrated and convenient approach that will greatly facilitate the genetic research of clonal species and double crosses.

Single-nucleotide polymorphism (SNP) markers are more and more often being used in genetic analysis. Liu *et al.* (2014) presented a HighMap method for constructing high-density linkage maps from next-generation sequencing (NGS). HighMap used an iterative ordering and error correction strategy based on a k-nearest neighbor algorithm and a Monte Carlo multipoint maximum likelihood algorithm, which also provided an idea for dealing with NGS data. Due to the bi-allelic characteristic, individual SNP markers cannot be in category I. However, any SNP marker can be category II, III, or IV in clonal F_1_, or category II, III, IV, or V in double crosses. In addition, by using the concept of haplotypes, it is possible to covert SNP markers to fully informative category I markers. For example, one haplotype is consisted of two closely linked SNP loci. Four genotypes can be identified by considering the two loci together, *i.e.*, 11, 10, 01, and 00. Then, the haplotype can be treated as category I marker in genetic analysis.

### Difference and similarity between clonal F_1_ and double cross

In clonal F_1_, genotype of the female parent can be either *A*_1_*B*_1_/*A*_2_*B*_2_ or *A*_1_*B*_2_/*B*_1_*A*_2_; genotype of the male parent can be either *C*_1_*D*_1_/*C*_2_*D*_2_ or *C*_1_*D*_2_/*D*_1_*C*_2_. In double cross, there are four homozygous inbred lines whose genotypes may be known. Alleles *A*, *B*, *C*, and *D* at each polymorphism locus can be traced back to the four inbred lines, when the four lines have been genotyped. In this case, genotype of the single cross between lines A and B is *A*_1_*B*_1_/*A*_2_*B*_2_; genotype of the single cross between lines C and D is *C*_1_*D*_1_/*C*_2_*D*_2_. Therefore, double cross is actually a special case of clonal F_1_ in which only linkage phase I is applicable (Figure S4).

In a double cross where polymorphism loci are only screened in the two single crosses, linkage phases become unknown before estimating recombination frequencies. Genotype of one single cross can be either *A*_1_*B*_1_/*A*_2_*B*_2_ or *A*_1_*B*_2_/*B*_1_*A*_2_; genotype of the other single cross can be either *C*_1_*D*_1_/*C*_2_*D*_2_ or *C*_1_*D*_2_/*D*_1_*C*_2_. In this case, the double cross must be treated as one clonal F_1_ population for genetic analysis (Figure S4), as is the case for the actual maize population used in this study.

Linkage phases in both parents of the clonal F_1_ can be determined by linkage analysis, from which four haploids can be built. If the four haploids could be viewed as haploids of the four inbred lines in a double cross, then clonal F_1_ is equivalent to double cross. In short, there are many similarities between clonal F_1_ and double cross, although difference does occur (Figure S4). It is important in genetics to know when clonal F_1_ and double cross are equivalent and when they are not. Previous genetic studies focused on only one of clonal F_1_ or double cross population. To our understanding, this study is the first that tried to combine the two kinds of populations. Based on the linkage analysis, two haploids of the female parent and two haploids of the male parent can be built, and then the clonal F_1_ progenies can be viewed as a double cross population derived from four inbred lines. The unified QTL mapping method for the two kinds of populations will be fully investigated in another article (Zhang *et al.* 2015).

### Classification of marker categories in clonal F_1_ and double cross

In clonal F_1_ and double crosses, both the number of identifiable alleles in parents and the number of identifiable genotypes in F_1_ progenies need to be considered in the classification of each marker locus. Wu *et al.* (2002a) only considered parents in marker classification, resulting in 18 possible cross types. However, many of them are identical in linkage analysis, and most cross types can be classified into the four marker categories in this study. For example, types A_1_ to A_4_ in Wu *et al.* (2002a) are identical to category I as defined in this study, because they all generate four genotypes that can be identified in the progenies.

Null alleles were also considered in Maliepaard *et al.* (1997) and Wu *et al.* (2002a, b). To our understanding, it is difficult to determine whether one parent carries two identical alleles or carries one allele and one null allele in practice. In the case of no missing data and no segregation distortion, type D_1_ in Wu *et al.* (2002a) can be decided by the 1:1 ratio test of two marker types in the progenies, and type A_3_ can be decided by the 1:1:1:1 ratio test of four marker types in the progenies. Unfortunately, missing data and segregation distortion are common in practical populations. In the case of type D_1_ and a large amount of missing marker points, we may wrongly say there are three or four marker classes. Even though we do know the number of marker type classes, the segregation ratio could be seriously affected by distortion. Therefore, we do not make the difference between cross types D_1_ and A_3_. Instead, both types were treated as nonpolymorphism in the male parent, *i.e.*, category III in this study.

### Wider applications of the clonal genetic analysis methods

In practice, clonal F_1_ progenies may come from the selfing pollination of one clonal parent, *i.e.*, female and male parents are from one clone population (Figure S4). In this case there are two alleles at each locus, and only marker category IV and linkage phases I and IV are applicable. Methods proposed in this study can be readily used to estimate recombination frequency, identify linkage phase, and build the two haploids of the clonal parent. In self-pollinated and cross-pollinated species, an F_2_ population is the selfing generation of one F_1_ hybrid between two inbred parents. Linkage phases are known when both inbred parents are genotyped. In this case, the clonal F_1_ derived from the selfing of one clonal parent can be viewed as an F_2_ population, after the two parental haploids are built.

If selfing can be viewed as a cross between the F_1_ hybrid and itself, the F_2_ population becomes a special case of clonal F_1_ when linkage phases are unknown, or a special case of double cross when linkage phases are known (Figure S4). In the F_2_ population, there are two alleles at each locus; therefore, only marker category IV is applicable. Haploids built in clonal F_1_ and double cross may help to identify and correct markers that are misclassified for the two inbred parents. Moreover, genetic analysis in an F_2_ population can still be performed by the clonal genetic analysis methods, even when there is no genotypic data on its two parental lines or on its F_1_ ancestry.

More broadly, methodology proposed in this study can be applied in genetic populations derived from any two heterozygotes in animals and plants. For example, in animals, linkage analysis is normally conducted in progenies between one female parent and one male parent, both are highly heterozygous, and they are drawn from a large random-mating population. By using the methodology of clonal F_1_, it is possible to build the female and male linkage maps to reflect the sex-specific recombination frequencies.

## Supplementary Material

Supporting Information
