# Supplementary material for: Linkage Analysis and Map Construction in Genetic Populations of Clonal F1 and Double Cross
Source: G3 (Bethesda). 2015 Jan 15;5(3):427–39. doi: 10.1534/g3.114.016022 (PMC4349096; doi:10.1534/g3.114.016022)
Supplement: Supporting Information [file supp_5_3_427__index.html]

Linkage Analysis and Map Construction in Genetic Populations of Clonal F1 and Double Cross — Supporting Information 

# Linkage Analysis and Map Construction in Genetic Populations of Clonal F1 and Double Cross

## Supporting Information for Zhang, Li, and Wang, 2015

**Files in this Data Supplement:**

- Supporting Information - Figures S1-S4, Files S1-S5, and Tables S1-S8 (PDF, 796 KB)
- Figure S1 - Diagram of the development of a double cross population from four inbred lines A, B, C, and D, which are highly homozygous at most loci. (PDF, 123 KB)
- Figure S2 - Five categories of polymorphism markers which can be used in genetic study in double cross populations. (PDF, 104 KB)
- Figure S3 - Combined, female, and male linkage maps of ten chromosomes in the actual maize population. (PDF, 820 KB)
- Figure S4 - Schematic representation on the difference and similarity between clonal F1 and double cross. (PDF, 111 KB)
- File S1 - Newton-Raphson algorithm in estimating *r* in Scenario 4 in clonal F1 progenies. (PDF, 172 KB)
- File S2 - Likelihood function, first and second order derivatives of the logarithm likelihood in estimating *r* in Scenario 9 for linkage phase I and IV in clonal F1 progenies. (PDF, 148 KB)
- File S3 - Main steps for the combined algorithm of nearest neighbor and Two-opt algorithm of Traveling Salesman Problem (TSP). (PDF, 134 KB)
- File S4 - Comparison with JoinMap, OneMap and R/qtl for linkage map construction in a simulated population with distorted markers. (PDF, 141 KB)
- File S5 - Comparison with JoinMap, OneMap and R/qtl for linkage map construction in a simulated clonal F1 population with 200 individuals and 200 markers belonging to Category IV. (PDF, 123 KB)
- Table S1 - Female and male gametes and their frequencies, and frequencies of their F1 progenies. (PDF, 106 KB)
- Table S2 - Theoretical frequencies of the twelve identifiable genotypes in the double cross population for Scenario 10. (PDF, 167 KB)
- Table S3 - Theoretical frequencies of the six identifiable genotypes in the double cross population for Scenarios 11 and 12. (PDF, 106 KB)
- Table S4 - Theoretical frequencies of the nine identifiable genotypes in the double cross population for Scenario 13 and 14. (PDF, 119 KB)
- Table S5 - Combined recombination frequencies between 20 markers in a simulated clonal F1 population. (PDF, 83 KB)
- Table S6 - Distance between the two markers (the upper triangular matrix) and LOD scores for detecting linkage (the lower triangular matrix) in the simulated clonal F1 population. (PDF, 84 KB)
- Table S7 - General information of the combined linkage maps of the two simulated populations with 20 markers built by GACD, JoinMap4.1, OneMap and R/qtl. (PDF, 82 KB)
- Table S8 - General information of the combined linkage maps of the simulated population with 200 individuals and 200 markers built by GACD, JoinMap4.1, OneMap and R/qtl. (PDF, 82 KB)
