# Supplementary material for: Linkage Analysis and Map Construction in Genetic Populations of Clonal F1 and Double Cross
Source: G3 (Bethesda). 2015 Jan 15;5(3):427–39. doi: 10.1534/g3.114.016022 (PMC4349096; doi:10.1534/g3.114.016022)
Supplement: Supporting Information [file supp_g3.114.016022_016022SI.pdf]

G3: Genes | Genomes | Genetics

Linkage Analysis and Map Construction in Genetic Populations of Clonal F_1 and Double Cross

Luyan Zhang, Huihui Li and Jiankang Wang*

The National Key Facility for Crop Gene Resources and Genetic Improvement, Institute of Crop Science, and CIMMYT China Office, Chinese Academy of Agricultural Sciences, Beijing 100081, China

***Corresponding Author:** Institute of Crop Science, Chinese Academy of Agricultural Sciences, No. 12 Zhongguancun South Street, Beijing 100081, China; e-mail: wangjiankang@caas.cn or jkwang@cgiar.org

DOI: 10.1534/g3.114.016022

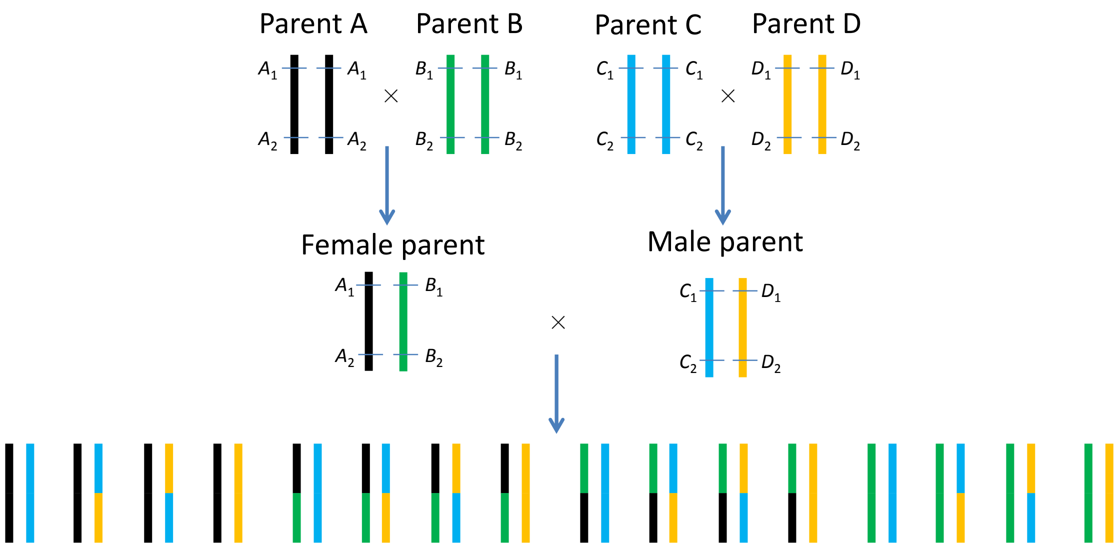

Figure S1 Diagram of the development of a double cross population from four inbred lines A, B, C, and D, which are highly homozygous at most loci. Assuming locus 1 and locus 2 were two linked polymorphism markers. A_1-D_1 were the four alleles at marker locus 1. A_2-D_2 were the four alleles at marker locus 2. Linkage phases in the two single crosses were known when the four inbred lines were genotyped.

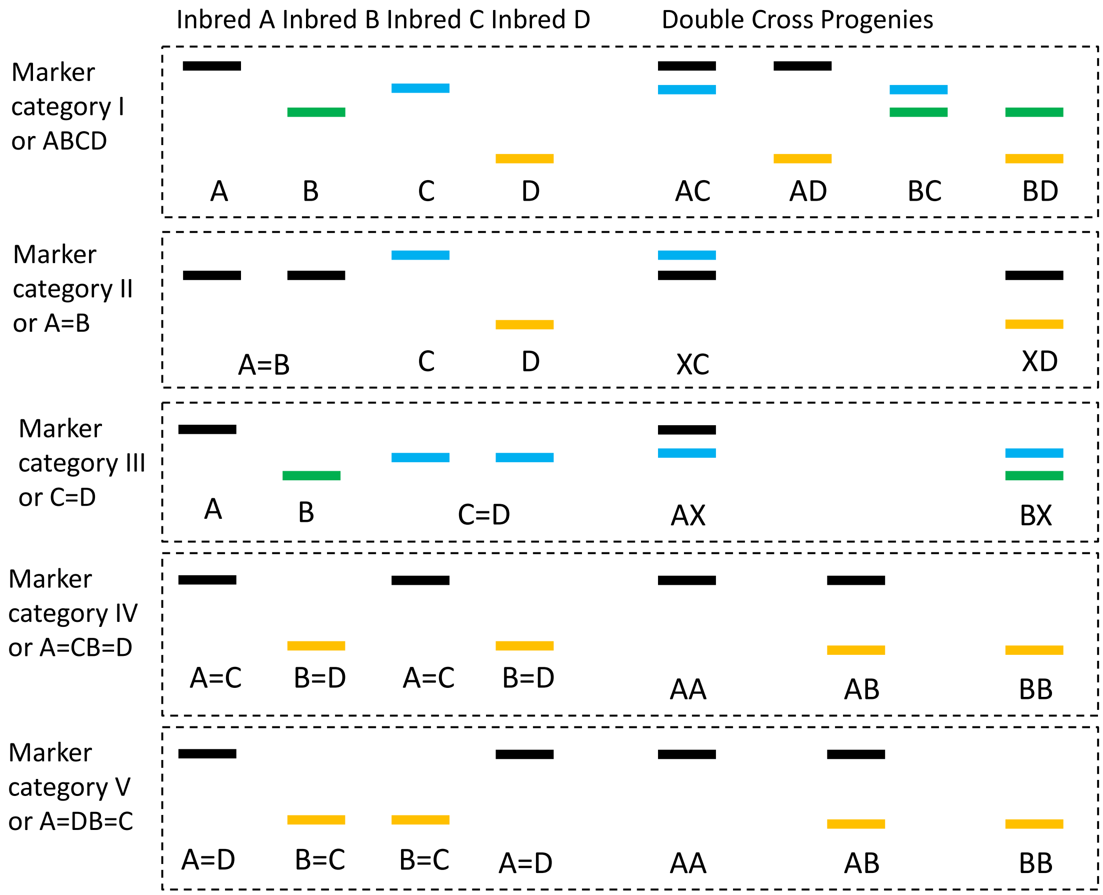

Figure S2 Five categories of polymorphism markers which can be used in genetic study in double cross populations. In Category I or ABCD, each marker shows four identifiable alleles between the four inbred parents, represented by *A*, *B*, *C* and *D* (see the four different colors in Figure S1). In the double cross population, four genotypes can be identified, represented by *AC*, *AD*, *BC* and *BD*. In Category II or A=B, one allele can be seen in parents *A* and *B*, and two alleles can be seen in parents *C* and *D*. In the double cross population, only two genotypes can be identified, represented by *XC* and *XD*, where *X* can be either *A* or *B*. In Category III or C=D, two alleles can be seen in parents A and B, and one allele can be seen in parents C and D. The two identifiable genotypes in the clonal population are represented by *AX* and *BX*, where *X* can be either *C* or *D*. In Category IV or A=CB=D, parents A and C show the same homozygous genotype, and parents *B* and *D* show the same homozygous genotype. The two alleles in four parents are represented by *A* and *B*, and three genotypes in their progenies are represented by *AA*, *AB* and *BB*. In Category V or A=DB=C, parents A and D show the same homozygous genotype, and parents B and C show the same homozygous genotype. The two alleles in four parents are represented by *A* and *B*, and three genotypes in their progenies are represented by *AA*, *AB* and *BB*.

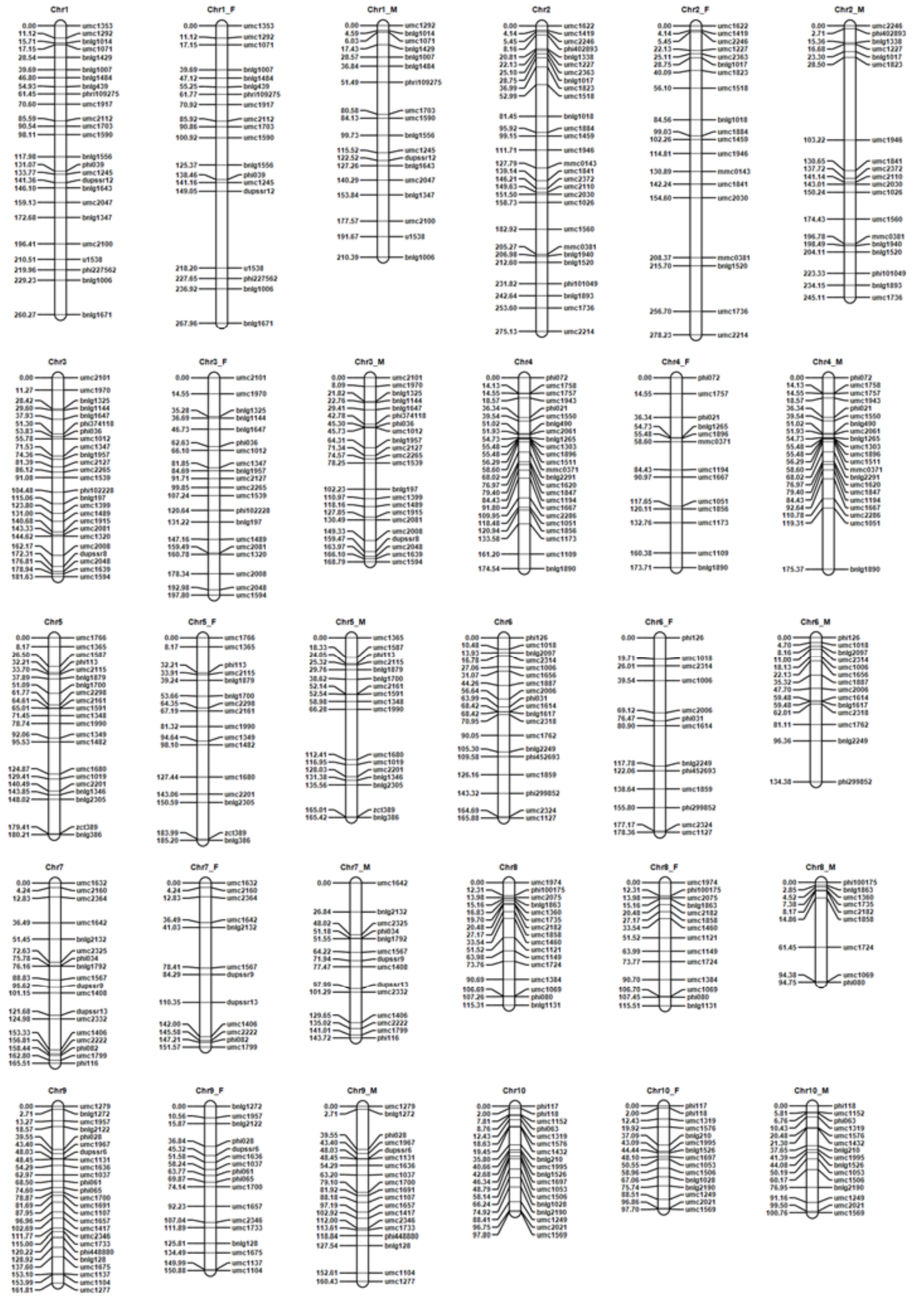

Figure S3 Combined, female, and male linkage maps of ten chromosomes in the actual maize population. Kosambi mapping function was used to convert recombination frequency to genetic distance.

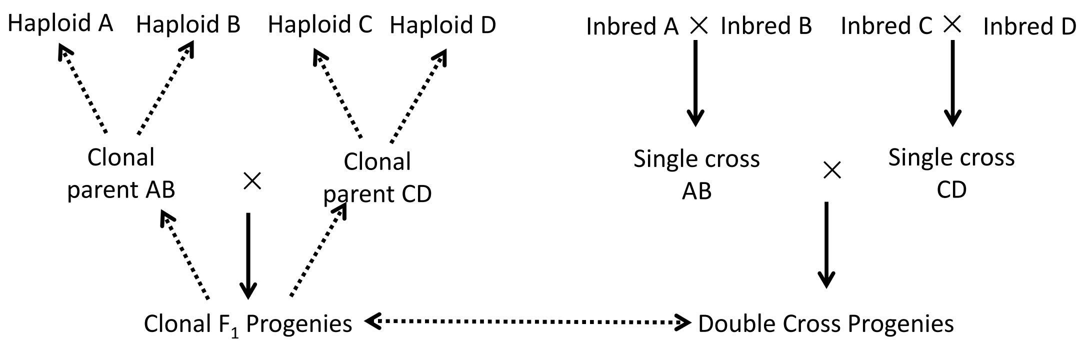

Figure S4 Schematic representation on the difference and similarity between clonal F_1 and double cross. In clonal F_1, the cross was made between two heterozygous parents. In double cross, the four parents were existing homozygous inbred lines. Two F_1 hybrids were firstly made from the four parents, and then the double cross was derived from the two F_1 hybrids. Solid arrows represent the derivative relationship in practice. In clonal F_1, haploids of the two parents can be built from the genetic analysis in its progenies, after which four virtual homozygous inbred lines may be determined. Dashed arrows stand for procedure where the four virtual lines were derived.

File S1

Newton-Raphson algorithm in estimating r in Scenario 4 in clonal F_1 progenies

For linkage phase I, using the theoretical frequencies of genotypes in Table 5, the likelihood function was given in Eq. (S1).

$$L = \frac{n!}{n_1!\cdots n_{12}!}\left[\frac{1}{4}(1-r)^2\right]^{n_1+n_{12}}\left[\frac{1}{4}r(1-r)\right]^{n_4+n_{6:7}+n_9}\left[\frac{1}{2}r(1-r)\right]^{n_2+n_{11}}\times$$

$$\left[\frac{1}{4}r^2\right]^{n_3+n_{10}}\left[\frac{1}{4}(1-2r+2r^2)\right]^{n_5+n_8}, \quad \text{(S1)}$$

where n_1-n_{12} were observed sample sizes of the 12 genotypes, $n_{i:j}$ is sum of n_i to n_j, and n was the total sample size (i.e. n=$n_{1:12}$). The logarithm of the likelihood was given in Eq. (S2).

$$\log L = C + (2n_1 + n_2 + n_4 + n_{6:7} + n_9 + n_{11} + 2n_{12})\log(1-r) +$$

$$(n_2 + 2n_3 + n_4 + n_{6:7} + n_9 + 2n_{10} + n_{11})\log r + (n_5 + n_8)\log(1-2r+2r^2) \quad \text{(S2)}$$

It is impossible to acquire an analytic MLE of *r* by solving the likelihood equation. Steps of Newton-Raphson algorithm to acquire a numerical solution of *r* were shown below.

Step 1: Assuming the initial value of *r* as 0.00001, and *ε* is the bearable error;

Step 2: Calculating the first derivative $f'(r)$ and the second derivative $f''(r)$ as given in Eq. S3 and S4, respectively.

$$f'(r) = \frac{d\ln L}{dr} = \frac{n_2 + 2n_3 + n_4 + n_{6:7} + n_9 + 2n_{10} + n_{11}}{r} -$$

$$\frac{2n_1 + n_2 + n_4 + n_{6:7} + n_9 + n_{11} + 2n_{12}}{1-r} + \frac{4r-2}{1-2r+2r^2}(n_5 + n_8) \quad \text{(S3)}$$

$$f''(r)=\frac{d^2\ln L}{d^2r}=-\frac{n_2+2n_3+n_4+n_{6:7}+n_9+2n_{10}+n_{11}}{r^2}-$$

$$\frac{2n_1+n_2+n_4+n_{6:7}+n_9+n_{11}+2n_{12}}{(1-r)^2}+\frac{8r(1-r)}{(1-2r+2r^2)^2}(n_5+n_8) \qquad \text{(S4)}$$

Step 3: Updating *r* as follows: $r_{i+1}=r_i-f^{'}(r_i)/f^{''}(r_i)$. If $|r_{i+1}-r_i|\le\varepsilon$ then let $\hat{r}=r_{i+1}$;

Otherwise, let $r_{i+1}=r_i$, and repeat step 2 until $|r_{i+1}-r_i|\le\varepsilon$.

For linkage phase II, the likelihood function and logarithm likelihood were given in Eq. S5 and S6, respectively.

$$L=\frac{n!}{n_1!\cdots n_{12}!}\left[\frac{1}{4}(1-r)^2\right]^{n_4+n_9}\left[\frac{1}{4}r(1-r)\right]^{n_1+n_3+n_{10}+n_{12}}\left[\frac{1}{2}r(1-r)\right]^{n_5+n_8}\times\cdot$$

$$\left[\frac{1}{4}r^2\right]^{n_{6:7}}\left[\frac{1}{4}(1-2r+2r^2)\right]^{n_2+n_{11}} \qquad \text{(S5)}$$

$$\log L=C+(n_1+n_3+2n_4+n_5+n_8+2n_9+n_{10}+n_{12})\log(1-r)+$$

$$(n_1+n_3+n_5+2n_{6:7}+n_8+2n_{10}+n_{12})\log r+(n_2+n_{11})\log(1-2r+2r^2) \qquad \text{(S6)}$$

The first derivative $f'(r)$ and the second derivative $f''(r)$ were given in Eq. S7 and S8, respectively.

$$f'(r)=\frac{d\ln L}{dr}=\frac{n_1+n_3+n_5+2n_{6:7}+n_8+n_{10}+n_{12}}{r}-+$$

$$\frac{n_1+n_3+2n_4+n_5+n_8+2n_9+n_{10}+n_{12}}{1-r}+\frac{4r-2}{1-2r+2r^2}(n_2+n_{11}) \qquad \text{(S7)}$$

$$f''(r)=\frac{d^2\ln L}{d^2r}=-\frac{n_1+n_3+n_5+2n_{6:7}+n_8+n_{10}+n_{12}}{r^2}-\cdot$$

$$\frac{n_1+n_3+2n_4+n_5+n_8+2n_9+n_{10}+n_{12}}{(1-r)^2}+\frac{8r(1-r)}{(1-2r+2r^2)^2}(n_2+n_{11}) \qquad \text{(S8)}$$

For linkage phase III, the likelihood function and logarithm likelihood were given in Eq. S9 and S10,

respectively.

$$L = \frac{n!}{n_1!\cdots n_{12}!}\left[\frac{1}{4}(1-r)^2\right]^{n_{6:7}}\left[\frac{1}{4}r(1-r)\right]^{n_1+n_3+n_{10}+n_{12}}\left[\frac{1}{2}r(1-r)\right]^{n_5+n_8}\times$$

$$\left[\frac{1}{4}r^2\right]^{n_4+n_9}\left[\frac{1}{4}(1-2r+2r^2)\right]^{n_2+n_{11}} \tag{S9}$$

The logarithm of the likelihood is therefore,

$$\log L = C + (n_1 + n_3 + n_5 + 2n_{6:7} + n_8 + 2n_{10} + n_{12})\log(1-r) +$$

$$(n_1 + n_3 + 2n_4 + n_5 + n_8 + 2n_9 + n_{10} + n_{12})\log r + (n_2 + n_{11})\log(1-2r+2r^2) \tag{S10}$$

The first derivative $f'(r)$ and the second derivative $f''(r)$ were given in Eq. S11 and S12, respectively.

$$f'(r) = \frac{d\ln L}{dr} = \frac{n_1 + n_3 + 2n_4 + n_5 + n_8 + 2n_9 + n_{10} + n_{12}}{r} -$$

$$\frac{n_1 + n_3 + n_5 + 2n_{6:7} + n_8 + n_{10} + n_{12}}{1-r} + \frac{4r-2}{1-2r+2r^2}(n_2 + n_{11}) \tag{S11}$$

$$f''(r) = \frac{d^2\ln L}{d^2 r} = -\frac{n_1 + n_3 + 2n_4 + n_5 + n_8 + 2n_9 + n_{10} + n_{12}}{r^2} -$$

$$\frac{n_1 + n_3 + n_5 + 2n_{6:7} + n_8 + n_{10} + n_{12}}{(1-r)^2} + \frac{8r(1-r)}{(1-2r+2r^2)^2}(n_2 + n_{11}) \tag{S12}$$

For linkage phase IV, the likelihood function and logarithm likelihood were given in Eq. S13 and S14, respectively.

$$L = \frac{n!}{n_1!\cdots n_{12}!}\left[\frac{1}{4}(1-r)^2\right]^{n_3+n_{10}}\left[\frac{1}{4}r(1-r)\right]^{n_4+n_{6:7}+n_9}\left[\frac{1}{2}r(1-r)\right]^{n_2+n_{11}}\times\cdot$$

$$\left[\frac{1}{4}r^2\right]^{n_1+n_{12}}\left[\frac{1}{4}\left(1-2r+2r^2\right)\right]^{n_5+n_8} \quad \text{(S13)}$$

$$\log L = C + (n_2 + 2n_3 + n_4 + n_{6:7} + n_9 + 2n_{10} + n_{11})\log(1-r) +$$

$$(2n_1 + n_2 + n_4 + n_{6:7} + n_9 + n_{11} + 2n_{12})\log r + (n_5 + n_8)\log(1-2r+2r^2) \quad \text{(S14)}$$

The first derivative $f'(r)$ and the second derivative $f''(r)$ were given in Eq. S15 and S16, respectively.

$$f'(r) = \frac{d\ln L}{dr} = \frac{2n_1 + n_2 + n_4 + n_{6:7} + n_9 + n_{11} + 2n_{12}}{r} -$$

$$\frac{n_2 + 2n_3 + n_4 + n_{6:7} + n_9 + 2n_{10} + n_{11}}{1-r} + \frac{4r-2}{1-2r+2r^2}\left(n_5 + n_8\right) \quad \text{(S15)}$$

$$f''(r) = \frac{d^2\ln L}{d^2 r} = -\frac{2n_1 + n_2 + n_4 + n_{6:7} + n_9 + n_{11} + 2n_{12}}{r^2} -$$

$$\frac{n_2 + 2n_3 + n_4 + n_{6:7} + n_9 + 2n_{10} + n_{11}}{\left(1-r\right)^2} + \frac{8r\left(1-r\right)}{\left(1-2r+2r^2\right)^2}\left(n_5 + n_8\right) \quad \text{(S16)}$$

File S2

Likelihood function, first and second order derivatives of the logarithm likelihood in estimating r in Scenario 9 for linkage phase I and IV in clonal F1 progenies

For linkage phase I, the likelihood function and logarithm likelihood were given in Eq. S17 and S18, respectively.

$$L = \frac{n!}{n_1!\cdots n_9!}\left[\frac{1}{4}(1-r)^2\right]^{n_1+n_9}\left[\frac{1}{2}r(1-r)\right]^{n_2+n_4+n_6+n_8}\left[\frac{1}{4}r^2\right]^{n_3+n_7}\left[\frac{1}{2}\left(1-2r+2r^2\right)\right]^{n_5} \quad \text{(S17)}$$

$$\log L = C + (2n_1+n_2+n_4+n_6+n_8+2n_9)\log(1-r) +$$

$$(n_2+2n_3+n_4+n_6+2n_7+n_8)\log r + n_5\log(1-2r+2r^2) \quad \text{(S18)}$$

where n_1-n_9 were observed sample sizes of the nine genotypes.

The first derivative $f'(r)$ and the second derivative $f''(r)$ were given in Eq. S19 and S20, respectively.

$$f'(r) = \frac{d\ln L}{dr} = \frac{n_2+2n_3+n_4+n_6+2n_7+n_8}{r} -$$

$$\frac{2n_1+n_2+n_4+n_6+n_8+2n_9}{1-r} + \frac{4r-2}{1-2r+2r^2}n_5 \quad \text{(S19)}$$

$$f''(r) = \frac{d^2\ln L}{d^2r} = -\frac{n_2+2n_3+n_4+n_6+2n_7+n_8}{r^2} -$$

$$\frac{2n_1+n_2+n_4+n_6+n_8+2n_9}{(1-r)^2} + \frac{8r(1-r)}{\left(1-2r+2r^2\right)^2}n_5 \quad \text{(S20)}$$

For linkage phase IV, the likelihood function and logarithm likelihood were given in Eq. S21 and S22, respectively.

$$L = \frac{n!}{n_1!\cdots n_9!}\left[\frac{1}{4}(1-r)^2\right]^{n_3+n_7}\left[\frac{1}{2}r(1-r)\right]^{n_2+n_4+n_6+n_8}\left[\frac{1}{4}r^2\right]^{n_1+n_9}\left[\frac{1}{2}\left(1-2r+2r^2\right)\right]^{n_5} \quad \text{(S21)}$$

$$\log L = C + (n_2 + 2n_3 + n_4 + n_6 + 2n_7 + n_8)\log(1-r) +$$

$$(2n_1 + n_2 + n_4 + n_6 + n_8 + 2n_9)\log r + n_5 \log(1 - 2r + 2r^2) \quad \text{(S22)}$$

The first derivative $f'(r)$ and the second derivative $f''(r)$ were given in Eq. S23 and S24, respectively.

$$f'(r) = \frac{d \ln L}{dr} = \frac{2n_1 + n_2 + n_4 + n_6 + n_8 + 2n_9}{r} -$$

$$\frac{n_2 + 2n_3 + n_4 + n_6 + 2n_7 + n_8}{1-r} + \frac{4r-2}{1-2r+2r^2} n_5 \quad \text{(S23)}$$

$$f''(r) = \frac{d^2 \ln L}{d^2 r} = -\frac{2n_1 + n_2 + n_4 + n_6 + n_8 + 2n_9}{r^2} -$$

$$\frac{n_2 + 2n_3 + n_4 + n_6 + 2n_7 + n_8}{(1-r)^2} + \frac{8r(1-r)}{(1-2r+2r^2)^2} n_5 \quad \text{(S24)}$$

File S3

Main steps for the combined algorithm of nearest neighbor and Two-opt algorithm of Traveling Salesman Problem (TSP)

Nearest neighbor algorithm was one of the first constructive algorithms to generate an initial solution of TSP. Its key idea was to visit the nearest neighbor each time. The main steps were as follows:

1. Select a city randomly.

2. Find the nearest unvisted city and go there.

3. If there are unvisited cities, repeat setp 2.

4. Return to the first city.

Although nearest neighbor algorithm was easy to understand and operate, it usually could not give the optimal solution for TSP. Then Two-opt algorithm was used for improving the solution. The main steps were as follows:

1. Generate a random initial solution T.

2. Select two cities i and k randomly and $i<k$. Then the modified solution was from city 1 to i-1, from city k reversely to i, and then from city k+1 to the end. If the modified solution can achieve a shorter length, the output solution was substuted by the modified solution.

3. If there are other pairs of i and k, repeat step 2.

4. Cut off the cycle from the longest interval.

File S4

Comparison with JoinMap, OneMap and R/qtl for linkage map construction in a simulated population with distorted markers

A simulated population with distorted markers was generated to investigate the effect of distortion on map construction. The original simulated population in Material and Methods was used as a start, among which none markers showed distortion. The steps to generate the distorted simulated population with distortion were as follows. Firstly, 50% marker points at Marker11 whose genotypes were *AC* or *AD* were set to be missing randomly. Then all individuals harboring missing genotypes at Marker 11 were deleted. In other words, the distorted population had only 150 individuals. Percentages of genotypes *AC*, *AD*, *BC* and *BD* at Marker11 were 16.7%, 19.3%, 33.3% and 30.7% whose P value of χ^2 test for segregation was 0.0068. Markers 6 to 9, 12, 14, 16 to 18 also showed segregation distortion under the significance level at 0.05.

General information of the combined linkage maps of the distorted population built by GACD, JoinMap4.1, OneMap and R/qtl were shown in Table S6. Similar to the original population, marker orders given by GACD, OneMap and R/qtl were the same as the predefined order. However, marker order given by JoinMap4.1 was far from predefined (Table S6). The true length was 100.13 cM. Lengths of the maps were 104.14 cM from GACD, 15203.82 cM from JoinMap, 102.92 cM from OneMap, and 104.38 cM from R/qtl. Time spent for building the maps was 6 s by GACD, 40 s by JoinMap, 334 s by OneMap, and 56 s by R/qtl. Distortion has little effect on linage map construction. Map lengths from GACD, OneMap and R/qtl were similar for this population, but OneMap and R/qtl had higher computational complexity and were much more time consuming than GACD.

File S5

Comparison with JoinMap, OneMap and R/qtl for linkage map construction in a simulated clonal F_1 population with 200 individuals and 200 markers belonging to Category IV

To investigate the effect of larger number markers on map construction, a simulated clonal F_1 population with 200 individuals and 200 markers was generated. All markers in this population belonged to Category IV. We considered one chromosome each with 200 evenly distributed markers. Recombination frequencies between any two neighboring markers were set at 0.099, equivalent to a genetic distance of 1.0 cM using Haldane mapping function. The length of the chromosome was 200 cM. For simplicity, one population with 200 bi-parental F_2 progenies was simulated by linkage map construction and QTL mapping software QTL IciMapping v4.0 (available from the web http://www.isbreeding.net/). This population could be regarded as a clonal F_1 population with all markers belonging to Category IV, or a double cross population with all markers belonging to Category V. Use GACD, JoinMap4.1, OneMap and R/qtl for map construction. All parameters for ordering markers were set as default.

General information of the combined linkage maps of this population built by GACD, JoinMap4.1, OneMap and R/qtl were listed in Table S7. R/qtl could not give results in the same personal computer owing to the computational complexity, and RGui showed the error message “Error: cannot allocate vector of size 438.4 Mb”. All markers could be linked to the chromosome by GACD. However, 5 and 24 markers could not be linked by JoinMap4.1 and OneMap respectively. Orders of all linked markers given by GACD, JoinMap4.1 and OneMap were the same as the predefined order. The true length was 200 cM. Lengths of the maps were 199.28 cM from GACD, 198.62 cM from JoinMap, and 198.18 cM from OneMap. Obviously, Length from GACD was the closest to the true length. Lengths from JoinMap and OneMap were a little shorter than GACD, which may be caused by the fewer number of markers on the chromosome. Time spent for building the maps was 0.5 min by GACD, 5 min by JoinMap, and 517 min by OneMap. Obviously, GACD gives the most accurate linkage map for this population using the shortest time.

Table S1 Female and male gametes and their frequencies, and frequencies of their F_1 progenies. Assuming four alleles can be clearly identified at each of the two linked loci. Genotypes of the female and male parents are A_1A_2/B_1B_2 and C_1D_2/C_1D_2, respectively. Recombination frequencies in the female and male parents are denoted as r_F and r_M, respectively.

Gamete and its frequency from the female F_1	Gamete and its frequency from the male F_1			
	C_1C_2, $\frac{1}{2}(1-r_M)$	C_1D_2, $\frac{1}{2}r_M$	D_1C_2, $\frac{1}{2}r_M$	D_1D_2, $\frac{1}{2}(1-r_M)$
A_1A_2, $\frac{1}{2}(1-r_F)$	$\frac{1}{4}(1-r_F)(1-r_M)$	$\frac{1}{4}(1-r_F)r_M$	$\frac{1}{4}(1-r_F)r_M$	$\frac{1}{4}(1-r_F)(1-r_M)$
A_1B_2, $\frac{1}{2}r_F$	$\frac{1}{4}r_F(1-r_M)$	$\frac{1}{4}r_Fr_M$	$\frac{1}{4}r_Fr_M$	$\frac{1}{4}r_F(1-r_M)$
B_1A_2, $\frac{1}{2}r_F$	$\frac{1}{4}r_F(1-r_M)$	$\frac{1}{4}r_Fr_M$	$\frac{1}{4}r_Fr_M$	$\frac{1}{4}r_F(1-r_M)$
B_1B_2, $\frac{1}{2}(1-r_F)$	$\frac{1}{4}(1-r_F)(1-r_M)$	$\frac{1}{4}(1-r_F)r_M$	$\frac{1}{4}(1-r_F)r_M$	$\frac{1}{4}(1-r_F)(1-r_M)$

Table S2 Theoretical frequencies of the twelve identifiable genotypes in the double cross population for Scenario 10. A_1, B_1, C_1 and D_1 are the four alleles at locus 1. A_2 and B_2 are the two alleles at locus 2. Recombination frequencies in the female and male parents are denoted as r_F and r_M, respectively. The combined recombination frequency is denoted as r. The last column gives the symbol of observed sample size of each genotype.

Genotype	Locus 1	Locus 2	Frequency	Combined recombination frequency	Sample size
1	A_1C_1	A_2A_2	$\frac{1}{4}(1-r_F)r_M$	$\frac{1}{4}r(1-r)$	n_1
2	A_1C_1	A_2B_2	$\frac{1}{4}(1-r_F)(1-r_M)+\frac{1}{4}r_F r_M$	$\frac{1}{4}(1-2r+2r^2)$	n_2
3	A_1C_1	B_2B_2	$\frac{1}{4}r_F(1-r_M)$	$\frac{1}{4}r(1-r)$	n_3
4	A_1D_1	A_2A_2	$\frac{1}{4}(1-r_F)(1-r_M)$	$\frac{1}{4}(1-r)^2$	n_4
5	A_1D_1	A_2B_2	$\frac{1}{4}(1-r_F)r_M+\frac{1}{4}r_F(1-r_M)$	$\frac{1}{2}r(1-r)$	n_5
6	A_1D_1	B_2B_2	$\frac{1}{4}r_F r_M$	$\frac{1}{4}r^2$	n_6
7	B_1C_1	A_2A_2	$\frac{1}{4}r_F r_M$	$\frac{1}{4}r^2$	n_7
8	B_1C_1	A_2B_2	$\frac{1}{4}(1-r_F)r_M+\frac{1}{4}r_F(1-r_M)$	$\frac{1}{2}r(1-r)$	n_8
9	B_1C_1	B_2B_2	$\frac{1}{4}(1-r_F)(1-r_M)$	$\frac{1}{4}(1-r)^2$	n_9
10	B_1D_1	A_2A_2	$\frac{1}{4}r_F(1-r_M)$	$\frac{1}{4}r(1-r)$	n_{10}
11	B_1D_1	A_2B_2	$\frac{1}{4}(1-r_F)(1-r_M)+\frac{1}{4}r_F r_M$	$\frac{1}{4}(1-2r+2r^2)$	n_{11}
12	B_1D_1	B_2B_2	$\frac{1}{4}(1-r_F)r_M$	$\frac{1}{4}r(1-r)$	n_{12}

Table S3 Theoretical frequencies of the six identifiable genotypes in the double cross population for Scenarios 11 and 12. For Scenario 11, X_1 (=A_1 or B_1), C_1 and D_1 are the three alleles at locus 1; A_2 and B_2 are the two alleles at locus 2. For Scenario 12, A_1, B_1, X_1 (=C_1 or D_1) are the three alleles at locus 1; A_2 and B_2 are the two alleles at locus 2. Recombination frequencies in the female and male parents are denoted as r_F and r_M, respectively. The last column gives the symbol of observed sample size of each genotype.

Genotype	Locus 1		Locus 2	Frequency		Sample size
	Scenario 11	Scenario 12		Scenario 11	Scenario 12	
1	X_1C_1	A_1X_1	A_2A_2	$\frac{1}{4}r_M$	$\frac{1}{4}(1-r_F)$	n_1
2	X_1C_1	A_1X_1	A_2B_2	$\frac{1}{4}$	$\frac{1}{4}$	n_2
3	X_1C_1	A_1X_1	B_2B_2	$\frac{1}{4}(1-r_M)$	$\frac{1}{4}r_F$	n_3
4	X_1D_1	B_1X_1	A_2A_2	$\frac{1}{4}(1-r_M)$	$\frac{1}{4}r_F$	n_4
5	X_1D_1	B_1X_1	A_2B_2	$\frac{1}{4}$	$\frac{1}{4}$	n_5
6	X_1D_1	B_1X_1	B_2B_2	$\frac{1}{4}r_M$	$\frac{1}{4}(1-r_F)$	n_6

Table S4 Theoretical frequencies of the nine identifiable genotypes in the double cross population for Scenario 13 and 14. A_1 and B_1 are the two alleles at locus 1; A_2 and B_2 are the two alleles at locus 2. The combined recombination frequency is denoted as r. The last column gives the symbol of observed sample size of each genotype.

Genotype	Locus 1	Locus 2	Expected frequency		Sample size
			Scenario 13	Scenario 14	
1	A_1A_1	A_2A_2	$\frac{1}{4}r(1-r)$	$\frac{1}{4}(1-r)^2$	n_1
2	A_1A_1	A_2B_2	$\frac{1}{4}(1-2r+2r^2)$	$\frac{1}{2}r(1-r)$	n_2
3	A_1A_1	B_2B_2	$\frac{1}{4}r(1-r)$	$\frac{1}{4}r^2$	n_3
4	A_1B_1	A_2A_2	$\frac{1}{4}(1-2r+2r^2)$	$\frac{1}{2}r(1-r)$	n_4
5	A_1B_1	A_2B_2	$r(1-r)$	$\frac{1}{2}(1-2r+2r^2)$	n_5
6	A_1B_1	B_2B_2	$\frac{1}{4}(1-2r+2r^2)$	$\frac{1}{2}r(1-r)$	n_6
7	B_1B_1	A_2A_2	$\frac{1}{4}r(1-r)$	$\frac{1}{4}r^2$	n_7
8	B_1B_1	A_2B_2	$\frac{1}{4}(1-2r+2r^2)$	$\frac{1}{2}r(1-r)$	n_8
9	B_1B_1	B_2B_2	$\frac{1}{4}r(1-r)$	$\frac{1}{4}(1-r)^2$	n_9

Table S5 Combined recombination frequencies between 20 markers in a simulated clonal F_1 population. The upper triangular was theoretical recombination frequencies, and the lower triangular matrix was estimated recombination frequencies.

Category	Marker	1	2	3	4	5	6	7	8	9	10	11	12	13	14	15	16	17	18	19	20
II	1		0.05	0.10	0.14	0.17	0.20	0.23	0.26	0.28	0.31	0.33	0.34	0.36	0.37	0.39	0.40	0.41	0.42	0.42	0.43
II	2	0.05		0.05	0.10	0.14	0.17	0.20	0.23	0.26	0.28	0.31	0.33	0.34	0.36	0.37	0.39	0.40	0.41	0.42	0.42
IV	3	0.06	0.04		0.05	0.10	0.14	0.17	0.20	0.23	0.26	0.28	0.31	0.33	0.34	0.36	0.37	0.39	0.40	0.41	0.42
III	4			0.08		0.05	0.10	0.14	0.17	0.20	0.23	0.26	0.28	0.31	0.33	0.34	0.36	0.37	0.39	0.40	0.41
III	5			0.11	0.03		0.05	0.10	0.14	0.17	0.20	0.23	0.26	0.28	0.31	0.33	0.34	0.36	0.37	0.39	0.40
IV	6	0.18	0.17	0.13	0.08	0.05		0.05	0.10	0.14	0.17	0.20	0.23	0.26	0.28	0.31	0.33	0.34	0.36	0.37	0.39
III	7			0.19	0.14	0.12	0.03		0.05	0.10	0.14	0.17	0.20	0.23	0.26	0.28	0.31	0.33	0.34	0.36	0.37
I	8	0.22	0.21	0.18	0.16	0.14	0.09	0.04		0.05	0.10	0.14	0.17	0.20	0.23	0.26	0.28	0.31	0.33	0.34	0.36
III	9			0.20	0.17	0.15	0.06	0.06	0.03		0.05	0.10	0.14	0.17	0.20	0.23	0.26	0.28	0.31	0.33	0.34
IV	10	0.26	0.23	0.23	0.20	0.18	0.16	0.08	0.09	0.05		0.05	0.10	0.14	0.17	0.20	0.23	0.26	0.28	0.31	0.33
I	11	0.31	0.30	0.26	0.24	0.22	0.18	0.13	0.12	0.08	0.05		0.05	0.10	0.14	0.17	0.20	0.23	0.26	0.28	0.31
IV	12	0.31	0.28	0.29	0.24	0.20	0.20	0.15	0.13	0.13	0.08	0.04		0.05	0.10	0.14	0.17	0.20	0.23	0.26	0.28
II	13	0.36	0.35	0.26			0.22		0.20		0.13	0.07	0.04		0.05	0.10	0.14	0.17	0.20	0.23	0.26
I	14	0.39	0.38	0.34	0.31	0.30	0.26	0.23	0.23	0.20	0.18	0.13	0.10	0.04		0.05	0.10	0.14	0.17	0.20	0.23
II	15	0.41	0.39	0.34			0.27		0.27		0.23	0.18	0.16	0.11	0.08		0.05	0.10	0.14	0.17	0.20
IV	16	0.45	0.44	0.39	0.38	0.37	0.30	0.25	0.28	0.24	0.26	0.21	0.17	0.15	0.09	0.06		0.05	0.10	0.14	0.17
I	17	0.44	0.42	0.40	0.36	0.35	0.34	0.28	0.30	0.26	0.29	0.26	0.22	0.22	0.15	0.13	0.07		0.05	0.10	0.14
III	18			0.37	0.35	0.34	0.30	0.27	0.27	0.26	0.26	0.24	0.20		0.09		0.05	0.04		0.05	0.10
I	19	0.48	0.46	0.45	0.37	0.36	0.37	0.31	0.35	0.30	0.34	0.31	0.29	0.32	0.23	0.25	0.17	0.12	0.07		0.05
II	20	0.49	0.47	0.48			0.41		0.42		0.36	0.35	0.29	0.32	0.32	0.26	0.23	0.17		0.08	

Table S6 Distance between the two markers (the upper triangular matrix) and LOD scores for detecting linkage (the lower triangular matrix) in the simulated clonal F_1 population. The distance was calculated using Haldane mapping function.

Category	Marker	1	2	3	4	5	6	7	8	9	10	11	12	13	14	15	16	17	18	19	20
II	1		5.3	6.5			22.6		29.0		37.4	47.1	48.9	61.9	73.5	83.0	116.1	102.0		160.9	175.3
II	2	43.0		4.2			20.8		27.2		31.1	44.6	41.0	58.6	69.3	73.5	107.0	88.6		126.3	133.0
IV	3	20.0	22.5		8.8	12.6	14.6	24.2	21.7	25.9	30.1	35.7	42.9	37.2	58.0	58.1	76.4	79.2	68.8	111.9	174.8
III	4			17.7		2.6	8.7	16.4	19.3	20.8	25.5	32.7	32.0		48.4		72.3	63.6	60.2	67.4	
III	5			14.8	50.1		4.8	13.1	15.7	17.1	22.2	28.1	26.3		44.6		68.3	58.6	55.4	61.9	
IV	6	8.4	9.0	32.4	15.9	19.4		3.5	10.2	6.0	20.0	21.8	26.2	28.3	36.9	39.4	46.8	55.8	44.7	66.0	85.2
III	7			8.8	25.0	29.2	20.8		4.2	6.4	9.2	15.1	17.9		30.8		35.6	41.0	38.8	48.4	
I	8	14.4	15.6	23.0	22.0	25.8	42.0	45.6		3.1	9.8	14.1	15.7	24.7	30.3	37.8	40.4	45.2	38.8	60.2	88.6
III	9			8.2	20.6	24.3	18.2	40.5	48.5		5.6	8.7	14.9		25.5		31.8	36.7	36.7	45.8	
IV	10	4.8	6.3	4.0	8.0	9.2	9.1	16.7	44.4	20.1		5.1	9.2	14.6	22.2	31.1	36.7	43.0	37.4	56.7	62.9
I	11	6.8	7.5	12.4	12.3	15.0	22.5	26.6	56.2	36.0	58.8		4.0	7.5	15.1	22.3	26.5	36.7	32.7	49.0	60.2
IV	12	2.9	4.1	1.4	5.9	7.6	5.4	10.9	31.7	12.5	0.0	63.6		4.5	10.8	19.5	21.1	29.5	26.3	43.4	43.5
II	13	3.7	4.2	5.1			6.6		17.4		13.0	38.2	20.8		4.2	12.4	17.4	29.0		49.7	51.1
I	14	2.3	2.7	4.9	6.4	7.5	10.9	13.4	27.3	16.7	22.4	53.6	41.1	45.6		8.7	9.8	17.1	9.9	30.3	51.1
II	15	1.6	2.3	2.1			4.1		10.0		6.3	19.3	10.2	30.1	36.0		6.3	15.1		33.7	36.7
IV	16	0.2	0.3	0.0	1.2	1.5	0.0	5.6	9.7	6.5	2.3	19.0	8.3	12.2	47.0	20.8		7.2	5.2	20.2	30.0
I	17	0.7	1.3	1.8	3.5	4.2	4.7	8.7	14.8	10.4	8.9	21.0	15.9	14.4	50.7	26.6	53.0		4.2	13.4	20.8
III	18			1.4	4.0	4.8	3.3	9.5	9.5	10.4	4.8	12.3	7.6		33.9		22.0	45.6		7.5	
I	19	0.1	0.3	1.0	3.0	3.7	3.5	6.4	8.6	7.2	5.0	13.0	8.9	6.1	30.3	11.9	26.5	57.6	38.2		8.1
II	20	0.0	0.2	0.0			0.6		1.3		1.7	4.0	3.7	5.8	5.8	10.4	7.1	20.6		37.1	

Table S7 General information of the combined linkage maps of the two simulated populations with 20 markers built by GACD, JoinMap4.1, OneMap and R/qtl.

Software	Order	Length (cM)	Time (s)
Original population			
GACD	1, 2, 3, 4, 5, 6, 7, 8, 9, 10, 11, 12, 13, 14, 15, 16, 17, 18, 19, 20	101.79	10
JoinMap4.1	12, 8, 5, 3, 4, 6, 7, 9, 14, 15, 20, 1, 2, 10, 11, 13, 17, 19, 16, 18	15211.04	33
OneMap	1, 2, 3, 4, 5, 6, 7, 8, 9, 10, 11, 12, 13, 14, 15, 16, 17, 18, 19, 20	103.83	455
R/qtl	1, 2, 3, 4, 5, 6, 7, 8, 9, 10, 11, 12, 13, 14, 15, 16, 17, 18, 19, 20	104.22	63
Distorted population			
GACD	1, 2, 3, 4, 5, 6, 7, 8, 9, 10, 11, 12, 13, 14, 15, 16, 17, 18, 19, 20	104.14	8
JoinMap4.1	12, 8, 5, 3, 4, 6, 7, 9, 14, 15, 20, 1, 2, 10, 11, 13, 17, 19, 16, 18	15203.82	45
OneMap	1, 2, 3, 4, 5, 6, 7, 8, 9, 10, 11, 12, 13, 14, 15, 16, 17, 18, 19, 20	102.92	334
R/qtl	1, 2, 3, 4, 5, 6, 7, 8, 9, 10, 11, 12, 13, 14, 15, 16, 17, 18, 19, 20	104.38	56

Table S8 General information of the combined linkage maps of the simulated population with 200 individuals and 200 markers built by GACD, JoinMap4.1, OneMap and R/qtl.

Software	No. of unlinked markers	No. of linked markers in correct order	First marker	Last marker	Length (cM)	Time (min)
GACD	0	200	Marker1	Marker200	199.28	0.5
JoinMap4.1	5	195	Marker1	Marker200	198.62	5
OneMap	24	176	Marker1	Marker198	198.18	537
R/qtl	-[a]	-	-	-	-	-

[a] R/qtl cannot give results for this population
