# Supplementary material for: Linkage Analysis and Map Construction in Genetic Populations of Clonal F1 and Double Cross
Source: G3 (Bethesda). 2015 Jan 15;5(3):427–39. doi: 10.1534/g3.114.016022 (PMC4349096; doi:10.1534/g3.114.016022)
Supplement: Supporting Information [file supp_g3.114.016022_FigureS1.pdf]

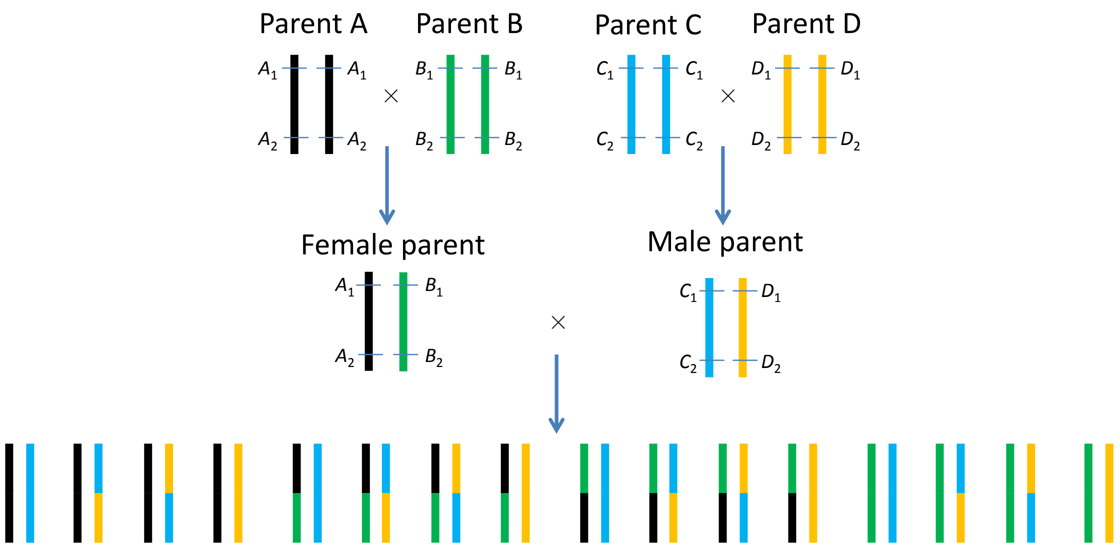

Figure S1 Diagram of the development of a double cross population from four inbred lines A, B, C, and D, which are highly homozygous at most loci. Assuming locus 1 and locus 2 were two linked polymorphism markers. A_1-D_1 were the four alleles at marker locus 1. A_2-D_2 were the four alleles at marker locus 2. Linkage phases in the two single crosses were known when the four inbred lines were genotyped.
