# Supplementary material for: Linkage Analysis and Map Construction in Genetic Populations of Clonal F1 and Double Cross
Source: G3 (Bethesda). 2015 Jan 15;5(3):427–39. doi: 10.1534/g3.114.016022 (PMC4349096; doi:10.1534/g3.114.016022)
Supplement: Supporting Information [file supp_g3.114.016022_FigureS2.pdf]

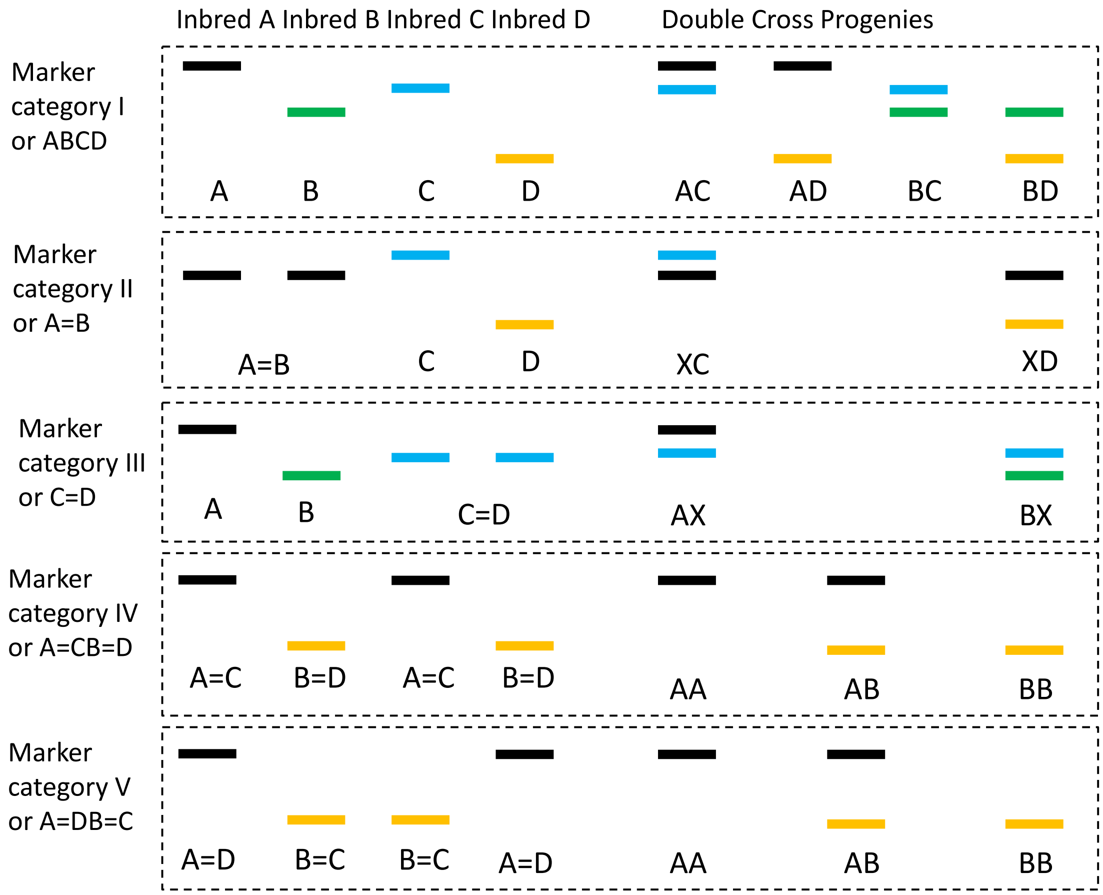

Figure S2 Five categories of polymorphism markers which can be used in genetic study in double cross populations. In Category I or ABCD, each marker shows four identifiable alleles between the four inbred parents, represented by *A*, *B*, *C* and *D* (see the four different colors in Figure S1). In the double cross population, four genotypes can be identified, represented by *AC*, *AD*, *BC* and *BD*. In Category II or A=B, one allele can be seen in parents *A* and *B*, and two alleles can be seen in parents *C* and *D*. In the double cross population, only two genotypes can be identified, represented by *XC* and *XD*, where *X* can be either *A* or *B*. In Category III or C=D, two alleles can be seen in parents A and B, and one allele can be seen in parents C and D. The two identifiable genotypes in the clonal population are represented by *AX* and *BX*, where *X* can be either *C* or *D*. In Category IV or A=CB=D, parents A and C show the same homozygous genotype, and parents *B* and *D* show the same homozygous genotype. The two alleles in four parents are represented by *A* and *B*, and three genotypes in their progenies are represented by *AA*, *AB* and *BB*. In Category V or A=DB=C, parents A and D show the same homozygous genotype, and parents B and C show the same homozygous genotype. The two alleles in four parents are represented by *A* and *B*, and three genotypes in their progenies are represented by *AA*, *AB* and *BB*.
