# Supplementary material for: Linkage Analysis and Map Construction in Genetic Populations of Clonal F1 and Double Cross
Source: G3 (Bethesda). 2015 Jan 15;5(3):427–39. doi: 10.1534/g3.114.016022 (PMC4349096; doi:10.1534/g3.114.016022)
Supplement: Supporting Information [file supp_g3.114.016022_FigureS3.pdf]

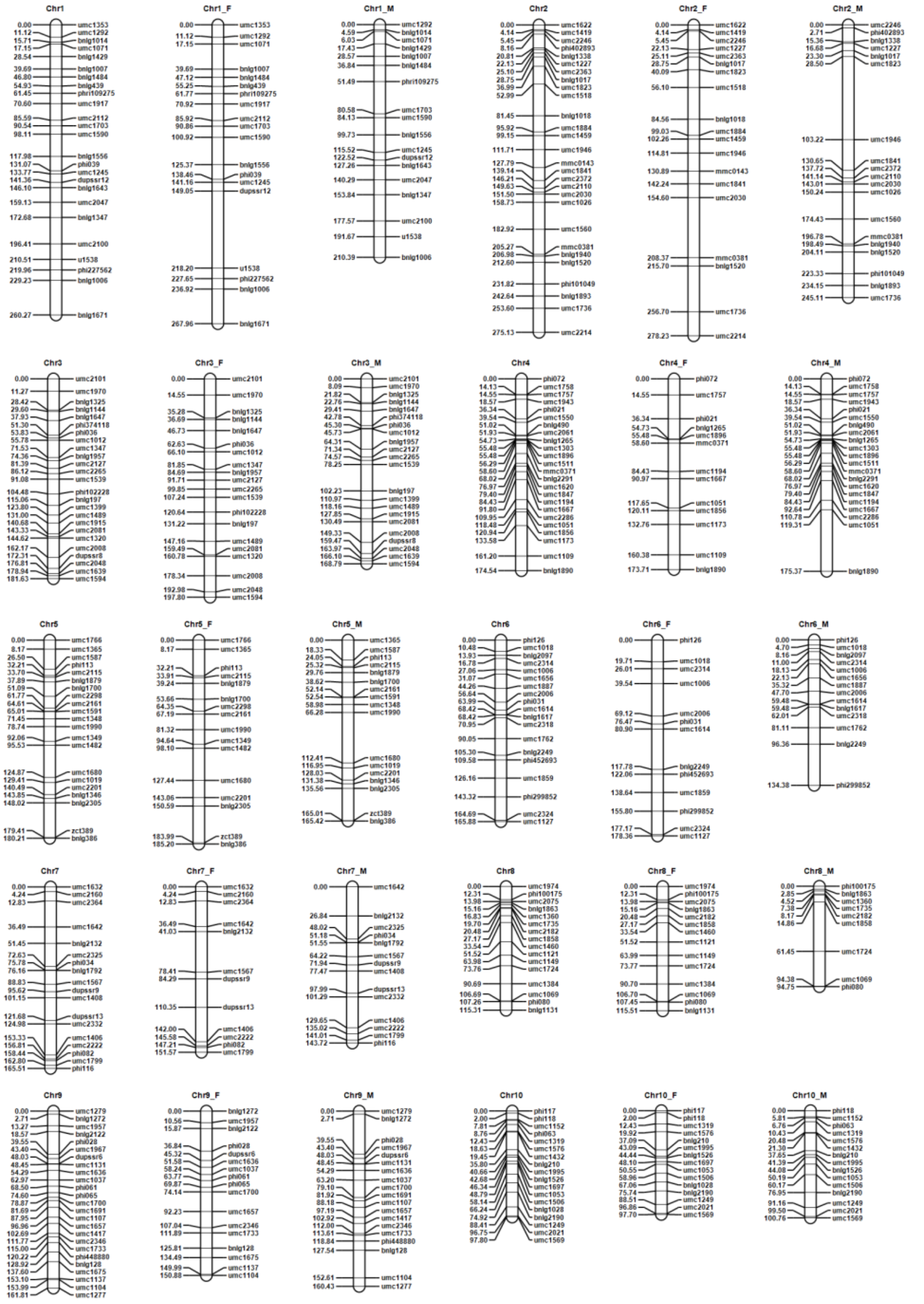

Figure S3 Combined, female, and male linkage maps of ten chromosomes in the actual maize population. Kosambi mapping function was used to convert recombination frequency to genetic distance.
