# Supplementary material for: Linkage Analysis and Map Construction in Genetic Populations of Clonal F1 and Double Cross
Source: G3 (Bethesda). 2015 Jan 15;5(3):427–39. doi: 10.1534/g3.114.016022 (PMC4349096; doi:10.1534/g3.114.016022)
Supplement: Supporting Information [file supp_g3.114.016022_FigureS4.pdf]

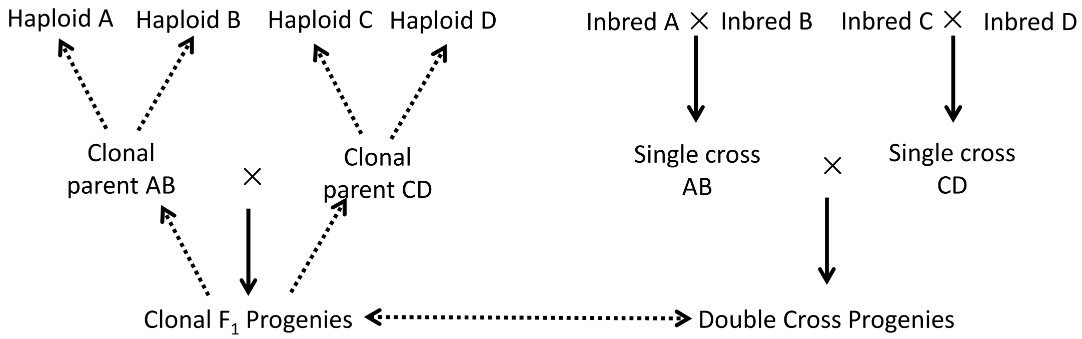

Figure S4 Schematic representation on the difference and similarity between clonal F_1 and double cross. In clonal F_1, the cross was made between two heterozygous parents. In double cross, the four parents were existing homozygous inbred lines. Two F_1 hybrids were firstly made from the four parents, and then the double cross was derived from the two F_1 hybrids. Solid arrows represent the derivative relationship in practice. In clonal F_1, haploids of the two parents can be built from the genetic analysis in its progenies, after which four virtual homozygous inbred lines may be determined. Dashed arrows stand for procedure where the four virtual lines were derived.
