# Supplementary material for: Linkage Analysis and Map Construction in Genetic Populations of Clonal F1 and Double Cross
Source: G3 (Bethesda). 2015 Jan 15;5(3):427–39. doi: 10.1534/g3.114.016022 (PMC4349096; doi:10.1534/g3.114.016022)
Supplement: Supporting Information [file supp_g3.114.016022_FileS1.pdf]

File S1

Newton-Raphson algorithm in estimating r in Scenario 4 in clonal F_1 progenies

For linkage phase I, using the theoretical frequencies of genotypes in Table 5, the likelihood function was given in Eq. (S1).

$$L = \frac{n!}{n_1!\cdots n_{12}!}\left[\frac{1}{4}(1-r)^2\right]^{n_1+n_{12}}\left[\frac{1}{4}r(1-r)\right]^{n_4+n_{6:7}+n_9}\left[\frac{1}{2}r(1-r)\right]^{n_2+n_{11}}\times$$

$$\left[\frac{1}{4}r^2\right]^{n_3+n_{10}}\left[\frac{1}{4}(1-2r+2r^2)\right]^{n_5+n_8}, \qquad \text{(S1)}$$

where n_1-n_{12} were observed sample sizes of the 12 genotypes, $n_{i:j}$ is sum of n_i to n_j, and n was the total sample size (i.e. n=$n_{1:12}$). The logarithm of the likelihood was given in Eq. (S2).

$$\log L = C + (2n_1 + n_2 + n_4 + n_{6:7} + n_9 + n_{11} + 2n_{12})\log(1-r) +$$

$$(n_2 + 2n_3 + n_4 + n_{6:7} + n_9 + 2n_{10} + n_{11})\log r + (n_5 + n_8)\log(1-2r+2r^2) \qquad \text{(S2)}$$

It is impossible to acquire an analytic MLE of *r* by solving the likelihood equation. Steps of Newton-Raphson algorithm to acquire a numerical solution of *r* were shown below.

Step 1: Assuming the initial value of *r* as 0.00001, and ε is the bearable error;

Step 2: Calculating the first derivative $f'(r)$ and the second derivative $f''(r)$ as given in Eq. S3 and S4, respectively.

$$f'(r) = \frac{d\ln L}{dr} = \frac{n_2 + 2n_3 + n_4 + n_{6:7} + n_9 + 2n_{10} + n_{11}}{r} -$$

$$\frac{2n_1 + n_2 + n_4 + n_{6:7} + n_9 + n_{11} + 2n_{12}}{1-r} + \frac{4r-2}{1-2r+2r^2}(n_5 + n_8) \qquad \text{(S3)}$$

$$f''(r) = \frac{d^2 \ln L}{d^2 r} = -\frac{n_2 + 2n_3 + n_4 + n_{6:7} + n_9 + 2n_{10} + n_{11}}{r^2} -$$

$$\frac{2n_1 + n_2 + n_4 + n_{6:7} + n_9 + n_{11} + 2n_{12}}{(1-r)^2} + \frac{8r(1-r)}{(1-2r+2r^2)^2}(n_5 + n_8) \tag{S4}$$

Step 3: Updating *r* as follows: $r_{i+1} = r_i - f'(r_i)/f''(r_i)$. If $|r_{i+1} - r_i| \le \varepsilon$ then let $\hat{r} = r_{i+1}$;

Otherwise, let $r_{i+1} = r_i$, and repeat step 2 until $|r_{i+1} - r_i| \le \varepsilon$.

For linkage phase II, the likelihood function and logarithm likelihood were given in Eq. S5 and S6, respectively.

$$L = \frac{n!}{n_1! \cdots n_{12}!}\left[\frac{1}{4}(1-r)^2\right]^{n_4+n_9}\left[\frac{1}{4}r(1-r)\right]^{n_1+n_3+n_{10}+n_{12}}\left[\frac{1}{2}r(1-r)\right]^{n_5+n_8} \times \cdot$$

$$\left[\frac{1}{4}r^2\right]^{n_{6:7}}\left[\frac{1}{4}(1-2r+2r^2)\right]^{n_2+n_{11}} \tag{S5}$$

$$\log L = C + (n_1 + n_3 + 2n_4 + n_5 + n_8 + 2n_9 + n_{10} + n_{12})\log(1-r) +$$

$$(n_1 + n_3 + n_5 + 2n_{6:7} + n_8 + 2n_{10} + n_{12})\log r + (n_2 + n_{11})\log(1-2r+2r^2) \tag{S6}$$

The first derivative $f'(r)$ and the second derivative $f''(r)$ were given in Eq. S7 and S8, respectively.

$$f'(r) = \frac{d \ln L}{dr} = \frac{n_1 + n_3 + n_5 + 2n_{6:7} + n_8 + n_{10} + n_{12}}{r} - +$$

$$\frac{n_1 + n_3 + 2n_4 + n_5 + n_8 + 2n_9 + n_{10} + n_{12}}{1-r} + \frac{4r-2}{1-2r+2r^2}(n_2 + n_{11}) \tag{S7}$$

$$f''(r) = \frac{d^2 \ln L}{d^2 r} = -\frac{n_1 + n_3 + n_5 + 2n_{6:7} + n_8 + n_{10} + n_{12}}{r^2} - \cdot$$

$$\frac{n_1 + n_3 + 2n_4 + n_5 + n_8 + 2n_9 + n_{10} + n_{12}}{(1-r)^2} + \frac{8r(1-r)}{(1-2r+2r^2)^2}(n_2 + n_{11}) \tag{S8}$$

For linkage phase III, the likelihood function and logarithm likelihood were given in Eq. S9 and S10,

respectively.

$$L = \frac{n!}{n_1!\cdots n_{12}!}\left[\frac{1}{4}(1-r)^2\right]^{n_{6:7}}\left[\frac{1}{4}r(1-r)\right]^{n_1+n_3+n_{10}+n_{12}}\left[\frac{1}{2}r(1-r)\right]^{n_5+n_8}\times$$

$$\left[\frac{1}{4}r^2\right]^{n_4+n_9}\left[\frac{1}{4}(1-2r+2r^2)\right]^{n_2+n_{11}} \quad \text{(S9)}$$

The logarithm of the likelihood is therefore,

$$\log L = C + (n_1+n_3+n_5+2n_{6:7}+n_8+2n_{10}+n_{12})\log(1-r) +$$

$$(n_1+n_3+2n_4+n_5+n_8+2n_9+n_{10}+n_{12})\log r + (n_2+n_{11})\log(1-2r+2r^2) \quad \text{(S10)}$$

The first derivative $f'(r)$ and the second derivative $f''(r)$ were given in Eq. S11 and S12, respectively.

$$f'(r) = \frac{d\ln L}{dr} = \frac{n_1+n_3+2n_4+n_5+n_8+2n_9+n_{10}+n_{12}}{r} -$$

$$\frac{n_1+n_3+n_5+2n_{6:7}+n_8+n_{10}+n_{12}}{1-r} + \frac{4r-2}{1-2r+2r^2}(n_2+n_{11}) \quad \text{(S11)}$$

$$f''(r) = \frac{d^2\ln L}{d^2 r} = -\frac{n_1+n_3+2n_4+n_5+n_8+2n_9+n_{10}+n_{12}}{r^2} -$$

$$\frac{n_1+n_3+n_5+2n_{6:7}+n_8+n_{10}+n_{12}}{(1-r)^2} + \frac{8r(1-r)}{(1-2r+2r^2)^2}(n_2+n_{11}) \quad \text{(S12)}$$

For linkage phase IV, the likelihood function and logarithm likelihood were given in Eq. S13 and S14, respectively.

$$L = \frac{n!}{n_1!\cdots n_{12}!}\left[\frac{1}{4}(1-r)^2\right]^{n_3+n_{10}}\left[\frac{1}{4}r(1-r)\right]^{n_4+n_{6:7}+n_9}\left[\frac{1}{2}r(1-r)\right]^{n_2+n_{11}}\times\cdot$$

$$\left[\frac{1}{4}r^2\right]^{n_1+n_{12}}\left[\frac{1}{4}\left(1-2r+2r^2\right)\right]^{n_5+n_8} \quad \text{(S13)}$$

$$\log L = C + (n_2 + 2n_3 + n_4 + n_{6:7} + n_9 + 2n_{10} + n_{11})\log(1-r) +$$

$$(2n_1 + n_2 + n_4 + n_{6:7} + n_9 + n_{11} + 2n_{12})\log r + (n_5 + n_8)\log(1-2r+2r^2) \quad \text{(S14)}$$

The first derivative $f'(r)$ and the second derivative $f''(r)$ were given in Eq. S15 and S16, respectively.

$$f'(r) = \frac{d\ln L}{dr} = \frac{2n_1 + n_2 + n_4 + n_{6:7} + n_9 + n_{11} + 2n_{12}}{r} -$$

$$\frac{n_2 + 2n_3 + n_4 + n_{6:7} + n_9 + 2n_{10} + n_{11}}{1-r} + \frac{4r-2}{1-2r+2r^2}\left(n_5 + n_8\right) \quad \text{(S15)}$$

$$f''(r) = \frac{d^2\ln L}{d^2r} = -\frac{2n_1 + n_2 + n_4 + n_{6:7} + n_9 + n_{11} + 2n_{12}}{r^2} -$$

$$\frac{n_2 + 2n_3 + n_4 + n_{6:7} + n_9 + 2n_{10} + n_{11}}{\left(1-r\right)^2} + \frac{8r\left(1-r\right)}{\left(1-2r+2r^2\right)^2}\left(n_5 + n_8\right) \quad \text{(S16)}$$
