# Supplementary material for: Linkage Analysis and Map Construction in Genetic Populations of Clonal F1 and Double Cross
Source: G3 (Bethesda). 2015 Jan 15;5(3):427–39. doi: 10.1534/g3.114.016022 (PMC4349096; doi:10.1534/g3.114.016022)
Supplement: Supporting Information [file supp_g3.114.016022_FileS2.pdf]

File S2

Likelihood function, first and second order derivatives of the logarithm likelihood in estimating r in Scenario 9 for linkage phase I and IV in clonal F1 progenies

For linkage phase I, the likelihood function and logarithm likelihood were given in Eq. S17 and S18, respectively.

$$L = \frac{n!}{n_1!\cdots n_9!}\left[\frac{1}{4}(1-r)^2\right]^{n_1+n_9}\left[\frac{1}{2}r(1-r)\right]^{n_2+n_4+n_6+n_8}\left[\frac{1}{4}r^2\right]^{n_3+n_7}\left[\frac{1}{2}\left(1-2r+2r^2\right)\right]^{n_5} \qquad \text{(S17)}$$

$$\log L = C + (2n_1 + n_2 + n_4 + n_6 + n_8 + 2n_9)\log(1-r) +$$

$$(n_2 + 2n_3 + n_4 + n_6 + 2n_7 + n_8)\log r + n_5 \log(1-2r+2r^2) \qquad \text{(S18)}$$

where n_1-n_9 were observed sample sizes of the nine genotypes.

The first derivative $f'(r)$ and the second derivative $f''(r)$ were given in Eq. S19 and S20, respectively.

$$f'(r) = \frac{d\ln L}{dr} = \frac{n_2 + 2n_3 + n_4 + n_6 + 2n_7 + n_8}{r} -$$

$$\frac{2n_1 + n_2 + n_4 + n_6 + n_8 + 2n_9}{1-r} + \frac{4r-2}{1-2r+2r^2}n_5 \qquad \text{(S19)}$$

$$f''(r) = \frac{d^2\ln L}{d^2 r} = -\frac{n_2 + 2n_3 + n_4 + n_6 + 2n_7 + n_8}{r^2} -$$

$$\frac{2n_1 + n_2 + n_4 + n_6 + n_8 + 2n_9}{(1-r)^2} + \frac{8r(1-r)}{\left(1-2r+2r^2\right)^2}n_5 \qquad \text{(S20)}$$

For linkage phase IV, the likelihood function and logarithm likelihood were given in Eq. S21 and S22, respectively.

$$L = \frac{n!}{n_1!\cdots n_9!}\left[\frac{1}{4}(1-r)^2\right]^{n_3+n_7}\left[\frac{1}{2}r(1-r)\right]^{n_2+n_4+n_6+n_8}\left[\frac{1}{4}r^2\right]^{n_1+n_9}\left[\frac{1}{2}\left(1-2r+2r^2\right)\right]^{n_5} \qquad \text{(S21)}$$

$$\log L = C + (n_2 + 2n_3 + n_4 + n_6 + 2n_7 + n_8)\log(1-r) +$$

$$(2n_1 + n_2 + n_4 + n_6 + n_8 + 2n_9)\log r + n_5 \log(1-2r+2r^2) \quad \text{(S22)}$$

The first derivative $f'(r)$ and the second derivative $f''(r)$ were given in Eq. S23 and S24, respectively.

$$f'(r) = \frac{d\ln L}{dr} = \frac{2n_1 + n_2 + n_4 + n_6 + n_8 + 2n_9}{r} -$$

$$\frac{n_2 + 2n_3 + n_4 + n_6 + 2n_7 + n_8}{1-r} + \frac{4r-2}{1-2r+2r^2} n_5 \quad \text{(S23)}$$

$$f''(r) = \frac{d^2 \ln L}{d^2 r} = -\frac{2n_1 + n_2 + n_4 + n_6 + n_8 + 2n_9}{r^2} -$$

$$\frac{n_2 + 2n_3 + n_4 + n_6 + 2n_7 + n_8}{(1-r)^2} + \frac{8r(1-r)}{(1-2r+2r^2)^2} n_5 \quad \text{(S24)}$$
