# Supplementary material for: Linkage Analysis and Map Construction in Genetic Populations of Clonal F1 and Double Cross
Source: G3 (Bethesda). 2015 Jan 15;5(3):427–39. doi: 10.1534/g3.114.016022 (PMC4349096; doi:10.1534/g3.114.016022)
Supplement: Supporting Information [file supp_g3.114.016022_FileS3.pdf]

File S3

Main steps for the combined algorithm of nearest neighbor and Two-opt algorithm of Traveling Salesman Problem (TSP)

Nearest neighbor algorithm was one of the first constructive algorithms to generate an initial solution of TSP. Its key idea was to visit the nearest neighbor each time. The main steps were as follows:

1. Select a city randomly.

2. Find the nearest unvisted city and go there.

3. If there are unvisited cities, repeat setp 2.

4. Return to the first city.

Although nearest neighbor algorithm was easy to understand and operate, it usually could not give the optimal solution for TSP. Then Two-opt algorithm was used for improving the solution. The main steps were as follows:

1. Generate a random initial solution T.

2. Select two cities i and k randomly and $i<k$. Then the modified solution was from city 1 to i-1, from city k reversely to i, and then from city k+1 to the end. If the modified solution can achieve a shorter length, the output solution was substuted by the modified solution.

3. If there are other pairs of i and k, repeat step 2.

4. Cut off the cycle from the longest interval.
