# Supplementary material for: Linkage Analysis and Map Construction in Genetic Populations of Clonal F1 and Double Cross
Source: G3 (Bethesda). 2015 Jan 15;5(3):427–39. doi: 10.1534/g3.114.016022 (PMC4349096; doi:10.1534/g3.114.016022)
Supplement: Supporting Information [file supp_g3.114.016022_FileS4.pdf]

File S4

Comparison with JoinMap, OneMap and R/qtl for linkage map construction in a simulated population with distorted markers

A simulated population with distorted markers was generated to investigate the effect of distortion on map construction. The original simulated population in Material and Methods was used as a start, among which none markers showed distortion. The steps to generate the distorted simulated population with distortion were as follows. Firstly, 50% marker points at Marker11 whose genotypes were *AC* or *AD* were set to be missing randomly. Then all individuals harboring missing genotypes at Marker 11 were deleted. In other words, the distorted population had only 150 individuals. Percentages of genotypes *AC*, *AD*, *BC* and *BD* at Marker11 were 16.7%, 19.3%, 33.3% and 30.7% whose P value of χ^2 test for segregation was 0.0068. Markers 6 to 9, 12, 14, 16 to 18 also showed segregation distortion under the significance level at 0.05.

General information of the combined linkage maps of the distorted population built by GACD, JoinMap4.1, OneMap and R/qtl were shown in Table S6. Similar to the original population, marker orders given by GACD, OneMap and R/qtl were the same as the predefined order. However, marker order given by JoinMap4.1 was far from predefined (Table S6). The true length was 100.13 cM. Lengths of the maps were 104.14 cM from GACD, 15203.82 cM from JoinMap, 102.92 cM from OneMap, and 104.38 cM from R/qtl. Time spent for building the maps was 6 s by GACD, 40 s by JoinMap, 334 s by OneMap, and 56 s by R/qtl. Distortion has little effect on linage map construction. Map lengths from GACD, OneMap and R/qtl were similar for this population, but OneMap and R/qtl had higher computational complexity and were much more time consuming than GACD.
