# Supplementary material for: Linkage Analysis and Map Construction in Genetic Populations of Clonal F1 and Double Cross
Source: G3 (Bethesda). 2015 Jan 15;5(3):427–39. doi: 10.1534/g3.114.016022 (PMC4349096; doi:10.1534/g3.114.016022)
Supplement: Supporting Information [file supp_g3.114.016022_FileS5.pdf]

File S5

Comparison with JoinMap, OneMap and R/qtl for linkage map construction in a simulated clonal F_1 population with 200 individuals and 200 markers belonging to Category IV

To investigate the effect of larger number markers on map construction, a simulated clonal F_1 population with 200 individuals and 200 markers was generated. All markers in this population belonged to Category IV. We considered one chromosome each with 200 evenly distributed markers. Recombination frequencies between any two neighboring markers were set at 0.099, equivalent to a genetic distance of 1.0 cM using Haldane mapping function. The length of the chromosome was 200 cM. For simplicity, one population with 200 bi-parental F_2 progenies was simulated by linkage map construction and QTL mapping software QTL IciMapping v4.0 (available from the web http://www.isbreeding.net/). This population could be regarded as a clonal F_1 population with all markers belonging to Category IV, or a double cross population with all markers belonging to Category V. Use GACD, JoinMap4.1, OneMap and R/qtl for map construction. All parameters for ordering markers were set as default.

General information of the combined linkage maps of this population built by GACD, JoinMap4.1, OneMap and R/qtl were listed in Table S7. R/qtl could not give results in the same personal computer owing to the computational complexity, and RGui showed the error message “Error: cannot allocate vector of size 438.4 Mb”. All markers could be linked to the chromosome by GACD. However, 5 and 24 markers could not be linked by JoinMap4.1 and OneMap respectively. Orders of all linked markers given by GACD, JoinMap4.1 and OneMap were the same as the predefined order. The true length was 200 cM. Lengths of the maps were 199.28 cM from GACD, 198.62 cM from JoinMap, and 198.18 cM from OneMap. Obviously, Length from GACD was the closest to the true length. Lengths from JoinMap and OneMap were a little shorter than GACD, which may be caused by the fewer number of markers on the chromosome. Time spent for building the maps was 0.5 min by GACD, 5 min by JoinMap, and 517 min by OneMap. Obviously, GACD gives the most accurate linkage map for this population using the shortest time.
