# Supplementary material for: Linkage Analysis and Map Construction in Genetic Populations of Clonal F1 and Double Cross
Source: G3 (Bethesda). 2015 Jan 15;5(3):427–39. doi: 10.1534/g3.114.016022 (PMC4349096; doi:10.1534/g3.114.016022)
Supplement: Supporting Information [file supp_g3.114.016022_TableS1.pdf]

Table S1 Female and male gametes and their frequencies, and frequencies of their F_1 progenies. Assuming four alleles can be clearly identified at each of the two linked loci. Genotypes of the female and male parents are A_1A_2/B_1B_2 and C_1D_2/C_1D_2, respectively. Recombination frequencies in the female and male parents are denoted as r_F and r_M, respectively.

Gamete and its frequency from the female F_1	Gamete and its frequency from the male F_1			
	C_1C_2, $\frac{1}{2}(1-r_M)$	C_1D_2, $\frac{1}{2}r_M$	D_1C_2, $\frac{1}{2}r_M$	D_1D_2, $\frac{1}{2}(1-r_M)$
A_1A_2, $\frac{1}{2}(1-r_F)$	$\frac{1}{4}(1-r_F)(1-r_M)$	$\frac{1}{4}(1-r_F)r_M$	$\frac{1}{4}(1-r_F)r_M$	$\frac{1}{4}(1-r_F)(1-r_M)$
A_1B_2, $\frac{1}{2}r_F$	$\frac{1}{4}r_F(1-r_M)$	$\frac{1}{4}r_Fr_M$	$\frac{1}{4}r_Fr_M$	$\frac{1}{4}r_F(1-r_M)$
B_1A_2, $\frac{1}{2}r_F$	$\frac{1}{4}r_F(1-r_M)$	$\frac{1}{4}r_Fr_M$	$\frac{1}{4}r_Fr_M$	$\frac{1}{4}r_F(1-r_M)$
B_1B_2, $\frac{1}{2}(1-r_F)$	$\frac{1}{4}(1-r_F)(1-r_M)$	$\frac{1}{4}(1-r_F)r_M$	$\frac{1}{4}(1-r_F)r_M$	$\frac{1}{4}(1-r_F)(1-r_M)$
