# Supplementary material for: Linkage Analysis and Map Construction in Genetic Populations of Clonal F1 and Double Cross
Source: G3 (Bethesda). 2015 Jan 15;5(3):427–39. doi: 10.1534/g3.114.016022 (PMC4349096; doi:10.1534/g3.114.016022)
Supplement: Supporting Information [file supp_g3.114.016022_TableS2.pdf]

**Table S2 Theoretical frequencies of the twelve identifiable genotypes in the double cross population for Scenario 10.** $A_1$, $B_1$, $C_1$ and $D_1$ are the four alleles at locus 1. $A_2$ and $B_2$ are the two alleles at locus 2. Recombination frequencies in the female and male parents are denoted as $r_F$ and $r_M$, respectively. The combined recombination frequency is denoted as $r$. The last column gives the symbol of observed sample size of each genotype.

| Genotype | Locus 1 | Locus 2 | Frequency | Combined recombination frequency | Sample size |
|---|---|---|---|---|---|
| 1 | $A_1C_1$ | $A_2A_2$ | $\frac{1}{4}(1-r_F)r_M$ | $\frac{1}{4}r(1-r)$ | $n_1$ |
| 2 | $A_1C_1$ | $A_2B_2$ | $\frac{1}{4}(1-r_F)(1-r_M)+\frac{1}{4}r_Fr_M$ | $\frac{1}{4}(1-2r+2r^2)$ | $n_2$ |
| 3 | $A_1C_1$ | $B_2B_2$ | $\frac{1}{4}r_F(1-r_M)$ | $\frac{1}{4}r(1-r)$ | $n_3$ |
| 4 | $A_1D_1$ | $A_2A_2$ | $\frac{1}{4}(1-r_F)(1-r_M)$ | $\frac{1}{4}(1-r)^2$ | $n_4$ |
| 5 | $A_1D_1$ | $A_2B_2$ | $\frac{1}{4}(1-r_F)r_M+\frac{1}{4}r_F(1-r_M)$ | $\frac{1}{2}r(1-r)$ | $n_5$ |
| 6 | $A_1D_1$ | $B_2B_2$ | $\frac{1}{4}r_Fr_M$ | $\frac{1}{4}r^2$ | $n_6$ |
| 7 | $B_1C_1$ | $A_2A_2$ | $\frac{1}{4}r_Fr_M$ | $\frac{1}{4}r^2$ | $n_7$ |
| 8 | $B_1C_1$ | $A_2B_2$ | $\frac{1}{4}(1-r_F)r_M+\frac{1}{4}r_F(1-r_M)$ | $\frac{1}{2}r(1-r)$ | $n_8$ |
| 9 | $B_1C_1$ | $B_2B_2$ | $\frac{1}{4}(1-r_F)(1-r_M)$ | $\frac{1}{4}(1-r)^2$ | $n_9$ |
| 10 | $B_1D_1$ | $A_2A_2$ | $\frac{1}{4}r_F(1-r_M)$ | $\frac{1}{4}r(1-r)$ | $n_{10}$ |
| 11 | $B_1D_1$ | $A_2B_2$ | $\frac{1}{4}(1-r_F)(1-r_M)+\frac{1}{4}r_Fr_M$ | $\frac{1}{4}(1-2r+2r^2)$ | $n_{11}$ |
| 12 | $B_1D_1$ | $B_2B_2$ | $\frac{1}{4}(1-r_F)r_M$ | $\frac{1}{4}r(1-r)$ | $n_{12}$ |
