# Supplementary material for: Linkage Analysis and Map Construction in Genetic Populations of Clonal F1 and Double Cross
Source: G3 (Bethesda). 2015 Jan 15;5(3):427–39. doi: 10.1534/g3.114.016022 (PMC4349096; doi:10.1534/g3.114.016022)
Supplement: Supporting Information [file supp_g3.114.016022_TableS3.pdf]

Table S3 Theoretical frequencies of the six identifiable genotypes in the double cross population for Scenarios 11 and 12. For Scenario 11, X_1 (=A_1 or B_1), C_1 and D_1 are the three alleles at locus 1; A_2 and B_2 are the two alleles at locus 2. For Scenario 12, A_1, B_1, X_1 (=C_1 or D_1) are the three alleles at locus 1; A_2 and B_2 are the two alleles at locus 2. Recombination frequencies in the female and male parents are denoted as r_F and r_M, respectively. The last column gives the symbol of observed sample size of each genotype.

Genotype	Locus 1		Locus 2	Frequency		Sample size
	Scenario 11	Scenario 12		Scenario 11	Scenario 12	
1	X_1C_1	A_1X_1	A_2A_2	$\frac{1}{4}r_M$	$\frac{1}{4}(1-r_F)$	n_1
2	X_1C_1	A_1X_1	A_2B_2	$\frac{1}{4}$	$\frac{1}{4}$	n_2
3	X_1C_1	A_1X_1	B_2B_2	$\frac{1}{4}(1-r_M)$	$\frac{1}{4}r_F$	n_3
4	X_1D_1	B_1X_1	A_2A_2	$\frac{1}{4}(1-r_M)$	$\frac{1}{4}r_F$	n_4
5	X_1D_1	B_1X_1	A_2B_2	$\frac{1}{4}$	$\frac{1}{4}$	n_5
6	X_1D_1	B_1X_1	B_2B_2	$\frac{1}{4}r_M$	$\frac{1}{4}(1-r_F)$	n_6
