# Supplementary material for: Linkage Analysis and Map Construction in Genetic Populations of Clonal F1 and Double Cross
Source: G3 (Bethesda). 2015 Jan 15;5(3):427–39. doi: 10.1534/g3.114.016022 (PMC4349096; doi:10.1534/g3.114.016022)
Supplement: Supporting Information [file supp_g3.114.016022_TableS4.pdf]

Table S4 Theoretical frequencies of the nine identifiable genotypes in the double cross population for Scenario 13 and 14. A_1 and B_1 are the two alleles at locus 1; A_2 and B_2 are the two alleles at locus 2. The combined recombination frequency is denoted as r. The last column gives the symbol of observed sample size of each genotype.

Genotype	Locus 1	Locus 2	Expected frequency		Sample size
			Scenario 13	Scenario 14	
1	A_1A_1	A_2A_2	$\frac{1}{4}r(1-r)$	$\frac{1}{4}(1-r)^2$	n_1
2	A_1A_1	A_2B_2	$\frac{1}{4}(1-2r+2r^2)$	$\frac{1}{2}r(1-r)$	n_2
3	A_1A_1	B_2B_2	$\frac{1}{4}r(1-r)$	$\frac{1}{4}r^2$	n_3
4	A_1B_1	A_2A_2	$\frac{1}{4}(1-2r+2r^2)$	$\frac{1}{2}r(1-r)$	n_4
5	A_1B_1	A_2B_2	$r(1-r)$	$\frac{1}{2}(1-2r+2r^2)$	n_5
6	A_1B_1	B_2B_2	$\frac{1}{4}(1-2r+2r^2)$	$\frac{1}{2}r(1-r)$	n_6
7	B_1B_1	A_2A_2	$\frac{1}{4}r(1-r)$	$\frac{1}{4}r^2$	n_7
8	B_1B_1	A_2B_2	$\frac{1}{4}(1-2r+2r^2)$	$\frac{1}{2}r(1-r)$	n_8
9	B_1B_1	B_2B_2	$\frac{1}{4}r(1-r)$	$\frac{1}{4}(1-r)^2$	n_9
