# Supplementary material for: Linkage Analysis and Map Construction in Genetic Populations of Clonal F1 and Double Cross
Source: G3 (Bethesda). 2015 Jan 15;5(3):427–39. doi: 10.1534/g3.114.016022 (PMC4349096; doi:10.1534/g3.114.016022)
Supplement: Supporting Information [file supp_g3.114.016022_TableS5.pdf]

Table S5 Combined recombination frequencies between 20 markers in a simulated clonal F_1 population. The upper triangular was theoretical recombination frequencies, and the lower triangular matrix was estimated recombination frequencies.

Category	Marker	1	2	3	4	5	6	7	8	9	10	11	12	13	14	15	16	17	18	19	20
II	1		0.05	0.10	0.14	0.17	0.20	0.23	0.26	0.28	0.31	0.33	0.34	0.36	0.37	0.39	0.40	0.41	0.42	0.42	0.43
II	2	0.05		0.05	0.10	0.14	0.17	0.20	0.23	0.26	0.28	0.31	0.33	0.34	0.36	0.37	0.39	0.40	0.41	0.42	0.42
IV	3	0.06	0.04		0.05	0.10	0.14	0.17	0.20	0.23	0.26	0.28	0.31	0.33	0.34	0.36	0.37	0.39	0.40	0.41	0.42
III	4			0.08		0.05	0.10	0.14	0.17	0.20	0.23	0.26	0.28	0.31	0.33	0.34	0.36	0.37	0.39	0.40	0.41
III	5			0.11	0.03		0.05	0.10	0.14	0.17	0.20	0.23	0.26	0.28	0.31	0.33	0.34	0.36	0.37	0.39	0.40
IV	6	0.18	0.17	0.13	0.08	0.05		0.05	0.10	0.14	0.17	0.20	0.23	0.26	0.28	0.31	0.33	0.34	0.36	0.37	0.39
III	7			0.19	0.14	0.12	0.03		0.05	0.10	0.14	0.17	0.20	0.23	0.26	0.28	0.31	0.33	0.34	0.36	0.37
I	8	0.22	0.21	0.18	0.16	0.14	0.09	0.04		0.05	0.10	0.14	0.17	0.20	0.23	0.26	0.28	0.31	0.33	0.34	0.36
III	9			0.20	0.17	0.15	0.06	0.06	0.03		0.05	0.10	0.14	0.17	0.20	0.23	0.26	0.28	0.31	0.33	0.34
IV	10	0.26	0.23	0.23	0.20	0.18	0.16	0.08	0.09	0.05		0.05	0.10	0.14	0.17	0.20	0.23	0.26	0.28	0.31	0.33
I	11	0.31	0.30	0.26	0.24	0.22	0.18	0.13	0.12	0.08	0.05		0.05	0.10	0.14	0.17	0.20	0.23	0.26	0.28	0.31
IV	12	0.31	0.28	0.29	0.24	0.20	0.20	0.15	0.13	0.13	0.08	0.04		0.05	0.10	0.14	0.17	0.20	0.23	0.26	0.28
II	13	0.36	0.35	0.26			0.22		0.20		0.13	0.07	0.04		0.05	0.10	0.14	0.17	0.20	0.23	0.26
I	14	0.39	0.38	0.34	0.31	0.30	0.26	0.23	0.23	0.20	0.18	0.13	0.10	0.04		0.05	0.10	0.14	0.17	0.20	0.23
II	15	0.41	0.39	0.34			0.27		0.27		0.23	0.18	0.16	0.11	0.08		0.05	0.10	0.14	0.17	0.20
IV	16	0.45	0.44	0.39	0.38	0.37	0.30	0.25	0.28	0.24	0.26	0.21	0.17	0.15	0.09	0.06		0.05	0.10	0.14	0.17
I	17	0.44	0.42	0.40	0.36	0.35	0.34	0.28	0.30	0.26	0.29	0.26	0.22	0.22	0.15	0.13	0.07		0.05	0.10	0.14
III	18			0.37	0.35	0.34	0.30	0.27	0.27	0.26	0.26	0.24	0.20		0.09		0.05	0.04		0.05	0.10
I	19	0.48	0.46	0.45	0.37	0.36	0.37	0.31	0.35	0.30	0.34	0.31	0.29	0.32	0.23	0.25	0.17	0.12	0.07		0.05
II	20	0.49	0.47	0.48			0.41		0.42		0.36	0.35	0.29	0.32	0.32	0.26	0.23	0.17		0.08	
