# Supplementary material for: Linkage Analysis and Map Construction in Genetic Populations of Clonal F1 and Double Cross
Source: G3 (Bethesda). 2015 Jan 15;5(3):427–39. doi: 10.1534/g3.114.016022 (PMC4349096; doi:10.1534/g3.114.016022)
Supplement: Supporting Information [file supp_g3.114.016022_TableS6.pdf]

Table S6 Distance between the two markers (the upper triangular matrix) and LOD scores for detecting linkage (the lower triangular matrix) in the simulated clonal F_1 population. The distance was calculated using Haldane mapping function.

Category	Marker	1	2	3	4	5	6	7	8	9	10	11	12	13	14	15	16	17	18	19	20
II	1		5.3	6.5			22.6		29.0		37.4	47.1	48.9	61.9	73.5	83.0	116.1	102.0		160.9	175.3
II	2	43.0		4.2			20.8		27.2		31.1	44.6	41.0	58.6	69.3	73.5	107.0	88.6		126.3	133.0
IV	3	20.0	22.5		8.8	12.6	14.6	24.2	21.7	25.9	30.1	35.7	42.9	37.2	58.0	58.1	76.4	79.2	68.8	111.9	174.8
III	4			17.7		2.6	8.7	16.4	19.3	20.8	25.5	32.7	32.0		48.4		72.3	63.6	60.2	67.4	
III	5			14.8	50.1		4.8	13.1	15.7	17.1	22.2	28.1	26.3		44.6		68.3	58.6	55.4	61.9	
IV	6	8.4	9.0	32.4	15.9	19.4		3.5	10.2	6.0	20.0	21.8	26.2	28.3	36.9	39.4	46.8	55.8	44.7	66.0	85.2
III	7			8.8	25.0	29.2	20.8		4.2	6.4	9.2	15.1	17.9		30.8		35.6	41.0	38.8	48.4	
I	8	14.4	15.6	23.0	22.0	25.8	42.0	45.6		3.1	9.8	14.1	15.7	24.7	30.3	37.8	40.4	45.2	38.8	60.2	88.6
III	9			8.2	20.6	24.3	18.2	40.5	48.5		5.6	8.7	14.9		25.5		31.8	36.7	36.7	45.8	
IV	10	4.8	6.3	4.0	8.0	9.2	9.1	16.7	44.4	20.1		5.1	9.2	14.6	22.2	31.1	36.7	43.0	37.4	56.7	62.9
I	11	6.8	7.5	12.4	12.3	15.0	22.5	26.6	56.2	36.0	58.8		4.0	7.5	15.1	22.3	26.5	36.7	32.7	49.0	60.2
IV	12	2.9	4.1	1.4	5.9	7.6	5.4	10.9	31.7	12.5	0.0	63.6		4.5	10.8	19.5	21.1	29.5	26.3	43.4	43.5
II	13	3.7	4.2	5.1			6.6		17.4		13.0	38.2	20.8		4.2	12.4	17.4	29.0		49.7	51.1
I	14	2.3	2.7	4.9	6.4	7.5	10.9	13.4	27.3	16.7	22.4	53.6	41.1	45.6		8.7	9.8	17.1	9.9	30.3	51.1
II	15	1.6	2.3	2.1			4.1		10.0		6.3	19.3	10.2	30.1	36.0		6.3	15.1		33.7	36.7
IV	16	0.2	0.3	0.0	1.2	1.5	0.0	5.6	9.7	6.5	2.3	19.0	8.3	12.2	47.0	20.8		7.2	5.2	20.2	30.0
I	17	0.7	1.3	1.8	3.5	4.2	4.7	8.7	14.8	10.4	8.9	21.0	15.9	14.4	50.7	26.6	53.0		4.2	13.4	20.8
III	18			1.4	4.0	4.8	3.3	9.5	9.5	10.4	4.8	12.3	7.6		33.9		22.0	45.6		7.5	
I	19	0.1	0.3	1.0	3.0	3.7	3.5	6.4	8.6	7.2	5.0	13.0	8.9	6.1	30.3	11.9	26.5	57.6	38.2		8.1
II	20	0.0	0.2	0.0			0.6		1.3		1.7	4.0	3.7	5.8	5.8	10.4	7.1	20.6		37.1	
