# Supplementary material for: Linkage Analysis and Map Construction in Genetic Populations of Clonal F1 and Double Cross
Source: G3 (Bethesda). 2015 Jan 15;5(3):427–39. doi: 10.1534/g3.114.016022 (PMC4349096; doi:10.1534/g3.114.016022)
Supplement: Supporting Information [file supp_g3.114.016022_TableS7.pdf]

Table S7 General information of the combined linkage maps of the two simulated populations with 20 markers built by GACD, JoinMap4.1, OneMap and R/qtl.

Software	Order	Length (cM)	Time (s)
Original population			
GACD	1, 2, 3, 4, 5, 6, 7, 8, 9, 10, 11, 12, 13, 14, 15, 16, 17, 18, 19, 20	101.79	10
JoinMap4.1	12, 8, 5, 3, 4, 6, 7, 9, 14, 15, 20, 1, 2, 10, 11, 13, 17, 19, 16, 18	15211.04	33
OneMap	1, 2, 3, 4, 5, 6, 7, 8, 9, 10, 11, 12, 13, 14, 15, 16, 17, 18, 19, 20	103.83	455
R/qtl	1, 2, 3, 4, 5, 6, 7, 8, 9, 10, 11, 12, 13, 14, 15, 16, 17, 18, 19, 20	104.22	63
Distorted population			
GACD	1, 2, 3, 4, 5, 6, 7, 8, 9, 10, 11, 12, 13, 14, 15, 16, 17, 18, 19, 20	104.14	8
JoinMap4.1	12, 8, 5, 3, 4, 6, 7, 9, 14, 15, 20, 1, 2, 10, 11, 13, 17, 19, 16, 18	15203.82	45
OneMap	1, 2, 3, 4, 5, 6, 7, 8, 9, 10, 11, 12, 13, 14, 15, 16, 17, 18, 19, 20	102.92	334
R/qtl	1, 2, 3, 4, 5, 6, 7, 8, 9, 10, 11, 12, 13, 14, 15, 16, 17, 18, 19, 20	104.38	56
