# Supplementary material for: Linkage Analysis and Map Construction in Genetic Populations of Clonal F1 and Double Cross
Source: G3 (Bethesda). 2015 Jan 15;5(3):427–39. doi: 10.1534/g3.114.016022 (PMC4349096; doi:10.1534/g3.114.016022)
Supplement: Supporting Information [file supp_g3.114.016022_TableS8.pdf]

Table S8 General information of the combined linkage maps of the simulated population with 200 individuals and 200 markers built by GACD, JoinMap4.1, OneMap and R/qtl.

Software	No. of unlinked markers	No. of linked markers in correct order	First marker	Last marker	Length (cM)	Time (min)
GACD	0	200	Marker1	Marker200	199.28	0.5
JoinMap4.1	5	195	Marker1	Marker200	198.62	5
OneMap	24	176	Marker1	Marker198	198.18	537
R/qtl	-[a]	-	-	-	-	-

[a] R/qtl cannot give results for this population
